# Design and application of virtual simulation teaching platform for intelligent manufacturing

**DOI:** 10.1038/s41598-024-62072-5

**Published:** 2024-06-05

**Authors:** Pengfei Zheng, Junkai Yang, Jingjing Lou, Bo Wang

**Affiliations:** 1 Xingzhi College, Zhejiang Normal University, Lanxi, 321100 China; 2 School of Mechanical Information, Yiwu Industrial & Commercial College, Yiwu, 322000 China; 3 School of Engineering, Huzhou University, Huzhou, 313000 China

**Keywords:** Online education, Computer technology, Intelligent manufacturing, Virtual simulation, Virtual-reality combination, Teaching platform, Information technology, Electrical and electronic engineering, Mechanical engineering

## Abstract

Aiming at the practical teaching of intelligent manufacturing majors faced with lack of equipment, tense teachers and other problems such as high equipment investment, high material loss, high teaching risk, difficult to implement internship, difficult to observe production, difficult to reproduce the results, and so on, we take the electrical automation technology, mechatronics technology and industrial robotics technology majors of intelligent manufacturing majors as an example, and design and establish a virtual simulation teaching platform for intelligent manufacturing majors by using the cloud computing platform, edge computing technology, and terminal equipment synergy. The platform includes six major virtual simulation modules, including virtual simulation of electrician electronics and PLC control, virtual and real combination of typical production lines of intelligent manufacturing, dual-axis collaborative robotics workstation, digital twin simulation, virtual disassembly of industrial robots, virtual simulation of magnetic yoke axis flexible production line. The platform covers the virtual simulation teaching content of basic principle experiments, advanced application experiments, and advanced integration experiments in intelligent manufacturing majors. In order to test the effectiveness of this virtual simulation platform for practical teaching in engineering, this paper organizes a teaching practice activity involving 246 students from two parallel classes of three different majors. Through a one-year teaching application, we analyzed the data on the grades of 7 core courses involved in three majors in one academic year, the proportion of participation in competitions and innovative activities, the number of awards and certificates of professional qualifications, and the subjective questionnaires of the testers. The analysis shows that the learners who adopt the virtual simulation teaching platform proposed in this paper for practical teaching are better than the learners under the traditional teaching method in terms of academic performance, proportion of participation in competitions and innovative activities, and proportion of awards and certificates by more than 13%, 37%, 36%, 27% and 22%, respectively. Therefore, the virtual simulation teaching platform of intelligent manufacturing established in this paper has obvious superiority in solving the problem of "three highs and three difficulties" existing in the practical teaching of engineering, and according to the questionnaire feedback from the testers, the platform can effectively alleviate the shortage of practical training equipment, stimulate the interest in learning, and help to broaden and improve the knowledge system of the learners.

## Introduction

Online education (E-learning) originated in the United States of America in the last century, and then emerged in the world, and gradually spread from North America, Europe and other regions to the Asian region. In the spring of 2020, a sudden epidemic of Covid-19 profoundly changed the way we take for granted how we live, work, and learn, and this epidemic also pushed online education into an unprecedented boom mode. It has been noted that the crisis has seriously affected the basic elements of higher education. Therefore, the way to achieve educational reconstruction during the epidemic was for students to use the Internet, television and other distance learning avenues for independent, cooperative, parent-guided and innovative learning. However, practical education in engineering, which is mainly based on experiments or hands-on training operations, is difficult to be solved by simply transplanting the generalized mode of online education, which is an important opportunity and challenge for the development of online education in engineering in the post-epidemic era and future.

For this reason, many scholars around the world have conducted extensive research on virtual simulation online education models or teaching platforms using artificial intelligence, virtual simulation, virtual reality, augmented reality and other technologies. Some researchers have conducted virtual simulation teaching and learning studies for high-risk types of experiments or courses in the fields of science, engineering, agriculture, medicine, and biochemistry. Krishnamoorthy^1^ reviewed the possibilities of using the existing online resources for teaching exclusively chemistry experiments virtually, and helped the teachers to teach exclusively the chemistry experiments and instruments remotely through virtual platforms. Chen et al.^2^ designed a psychological virtual simulation experimental teaching system to solve the problem of “invisibility” of human psychological activities. Kruger et al.^3^ proposed integrating the benefits of the different modes in the context of control system learning in a mechatronics engineering course, and designed a low‐cost ball‐on‐beal demonstrator with Matlab/Simulink software interface to bridge the theory‐practice divide in teaching state space control theory. Elkhatat et al.^4^ used the computer‐based Aspen Plus® Sensitivity Analysis Tool (APSAT) to establish a virtual environment to mimic a gas absorption lab experiment in the Unit Operations Lab within the curriculum for the Bachelor of Science in Chemical Engineering. Nadeem et al.^5^ presented a mobile application aimed at novice learners that makes use of technology for the teaching and learning of computer system engineering concepts. Lamo et al.^6^ presented the case of study of a flexible laboratory for the use of Arduino in a Computer Technology course with 153 students, geographically distributed in Spain and Latin America. Lai et al.^7^ designed a three-dimensional integrated circuit virtual experiment platform based on Unity3d, which used the Unity3d and 3ds Max tools to build a three-dimensional model of instruments, equipment, electronic components, and ultra cleanroom laboratory scenes in an integrated circuit experiment. Lionetti et al.^8^ studied two Engaging Options for teaching microscopy remotely. Sreekanth et al.^9^ proposed an innovative teaching–learning system that helps conduct chemistry volumetric experiments through a hardware-enabled platform named avatar-shell. Zheng et al.^10^ explained the design, development and application of virtual simulation teaching resources of "Safe Electricity" based on Unity3D. Reen et al.^11^ explored the integration of an immersive virtual reality simulation based on a challenging molecular biology concept within an existing module taught at University College Cork. Additionally, some other scholars have established virtual simulation systems for some high-cost application environments or scenarios, such as transportation, water conservancy engineering, earthquake and disaster prevention, and robotic systems. Dong et al.^12^ established a virtual simulation experiment teaching platform by 3D modeling, human–computer interaction, and VR technologies. Their platform allowed students to understand the structure of the subway ventilation room, and master the control requirements of the ventilation system in the event of sudden fire, blockage, and failure in the subway. Rajabi et al.^13^ evaluated the effect of education and premonition on the incorrect decision-making of residents under the stressful conditions of an earthquake, and then designed a virtual model based on a proposed classroom in a school in the city of Tehran to simulate a virtual learning experience. Pang et al.^14^ built an online remote robotics experiment system using digital twin (DT) technology and IoT technology and adopted ADDIE (Analysis, Design, Development, Implementation, and Evaluation) teaching method. Zhao et al.^15^ developed a virtual simulation cloud system of Waterway Engineering Design based on outcome-based education. Cho et al.^16^ proposed an immersive virtual reality simulation for environmental education based on the virtual ecosystem model and two applications based on this simulation. Jayasundera et al.^17^ indicated that virtual reality simulation (VRS) supports students’ transition back to patient care by increasing post-intervention confidence in clinical decision making, management, and patient communication. Nevertheless, other researchers have analyzed and summarized the effectiveness of virtual simulation technology applied to teaching and learning. Fernández et al.^18^ developed a platform and protocol (LICIEXAM) for the in‐advance seat reservation and simultaneous online and in‐person class attendance, and examination tools and strategies, with a special emphasis on avoiding online cheating. Cerra et al.^19^ showed the advantages of integrating interactive self‐assessment tools into CAD learning methodologies, such as problem‐based learning (PBL). Vergara et al.^20^ carried out a quantitative research on the player profile and the one considered best for learning engineering professors, and identified the sociological and academic aspects that influence player profile choices, then designed a survey for a descriptive and inferential analysis of the answers given by a population of 532 engineering professors. Xie et al.^21^ constructed a conceptual model and structural equation model (SEM) to analyze the virtual simulation experiment teaching effect by the SEM analytical method. Davis et al.^22^ examined a model of the impact of perceptual variables on instructional effectiveness that can enhance teaching efficacy and outcome expectancy when preservice teachers engage in practice teaching experiences in a virtual classroom simulation. Ke et al.^23^ indicated that the VR simulation better promoted the lab-teaching knowledge development than the live simulation, whereas the latter better fostered class-teaching knowledge development. Li et al.^24^ discussed the efficacy of virtual simulation system teaching method in improving critical thinking of engineering students engaged in NC (Numerical Control) machining. In order to solve the problems of poor user experience and lack of navigational guidance in virtual simulation pedagogy, Dong et al.^25^ proposed a scheme for establishing an intelligent navigational chemical laboratory based on multimodal fusion. Khalilia et al.^26^ concluded that the learning process is supported through the implementation and study of modern learning strategies and activities for teaching crime scene investigation using virtual reality technology. An improved teaching mechanism empowered by edge computing–driven VR was proposed to enhance the education experience and improve the teaching environment^27^. Lu et al.^28^ built an experimental teaching platform using virtual simulation technology for vital pulpotomy that includes learning and examination modes, which can effectively improve the teaching of pulpotomy. Zulfiqar et al.^29^ discussed the concept of AR and its types, the need for AR applications in education, analysis of various state-of-the-art AR applications in terms of platform, augmented virtual content, interactions, usability, usefulness, performance, effectiveness, and ease-of-use under a single taxonomy, they gave a more comprehensive summary of the applications of AR technology in education and teaching. Similary, Chen et al.^30^ gave a review of Artificial Intelligence (AI) in education, and assessed the impact of AI on education. Homoplastically, some researchers have conducted in-depth studies on assembly operation environment simulation using virtual reality, augmented reality and other technologies, and applied them to training, workshop analysis and design scenarios of assembly operation with good results. Dimitropoulos et al.^31^ proposed a flexible framework that provided the necessary tools and functionalities allowing non expert users to create 1:1 replicas of industrial shop floors in a non-time-consuming way. Apostolopoulos et al.^32^ presented a novel operator training framework based on Augmented Reality (AR) technology. Michalos et al.^33^ proposed a method to analyze and enhance industrial workplaces using immersive virtual reality. Aivaliotis et al.^34^ and Kousi et al.^35^ presented an augmented reality software suite aiming at supporting the operator's interaction with flexible mobile robot workers. Other scholars discussed the applications of digital twin technology to robotic assembly lines^36–38^. The virtual simulation practice teaching platform involves many disciplines, and the majority of electronic information and mechanical engineering, we categorized each of the above virtual simulation teaching platforms, as listed in Table 1.

**Table1:** Classification of virtual simulation practice teaching platforms.

Virtual platforms	Subject classification or course type	Description
Ref. ^1,4,9,25^	Chemistry & Chemical	Basic chemistry experiments, Gas absorption experiments, Chemistry volumetric experiments, etc
Ref. ^2,8,11,13,17,28^	Biomedicine and Psychology	Mental processing and cognition simulation experiments, Earthquakes feeling experiments, Clinical decision-making experiments, Molecular biology experiments, etc
Ref. ^3,5–7,10,18^	Electronic & Informatics	Ball-on-beam experiments, Electronic circuit experiments, Safe electricity experiments, Arduino board experiments, etc
Ref. ^14,19,20,24,31–33^	Mechanical Engineering	Robot programming experiments, CAD pedagogy experiments, NC machining experiments, Assembly operation experiments, etc
Ref. ^12,15,16^	Construction and Environmental Engineering	Subway emergency ventilation experiments, Waterway engineering design experiments, Ecosystem model experiments, etc

However, there are fewer studies on basic virtual simulation public teaching platforms designed for a major category of specialties, such as chemical and chemical industry, biomedicine, aerospace, intelligent manufacturing, and so on. Therefore, in order to explore the new ideas, new modes and new mechanisms of virtual simulation-enabled experimental teaching, to help upgrade the digitalization of education, to cope with the changes in the practical training teaching environment of engineering majors, and to build a new model of practical teaching in science and engineering, this paper proposes a research on the construction of a virtual-real combination of practical training teaching platform under the three-body synergistic architecture of cloud computing, edge computing, and mobile terminal technology. For the basic content in the intelligent manufacturing class of specialties, the use of virtual simulation technology to design three-dimensional virtual wiring, PLC control, electrical signal monitoring and other modules, to achieve the electrical and electronic, PLC control class of basic experiments in the virtual and real combination of practical training teaching; For the higher-order applications in intelligent manufacturing majors, based on the form of industry-teaching fusion and school-enterprise cooperation, we develop the virtual simulation teaching module of automation production line based on the actual production and introduce it into the cloud training platform to realize the virtual simulation teaching of higher-order experiments in the intelligent control, which can be used to serve the training of the students in school and the employees of the enterprises; For the problems of lack of experimental equipment and tense workstations in engineering, the cloud practical training teaching platform is designed to meet the learning needs of students such as multi-location, remote, and no time limit, and to provide a new way of thinking for the application of online education to practical courses.

**Figure 1:**
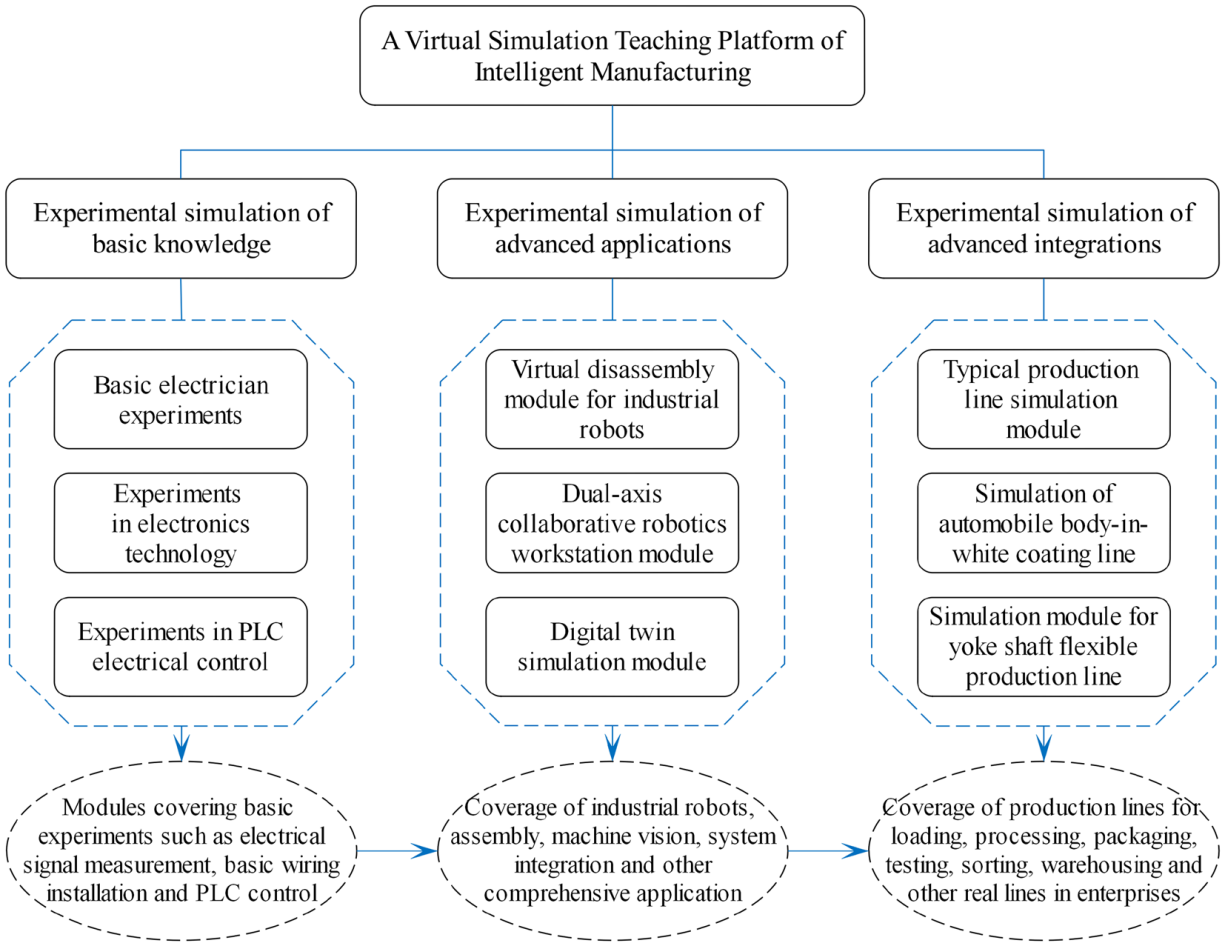
Architecture of the virtual simulation teaching platform.

The remainder of this paper is organized as follows: Section "Overall platform design" constructs a "cloud, edge, end" three-body synergistic combination of virtual and real simulation teaching platform, which includes electrician electronics and PLC control virtual simulation, intelligent manufacturing typical production line virtual and real combination, two-axis collaborative robotics workstations, digital twin simulation, virtual disassembly of industrial robots, and the magnetic yoke axis flexible production line virtual simulation and other modules. Section "Applications and discussion" discusses the practice of the simulation teaching platform, and its teaching effect and evaluation. In Section "Conclusions", we show the conclusions and the future of this study, and the overall frame is shown in Fig. 1.

## Overall platform design

**Figure 2:**
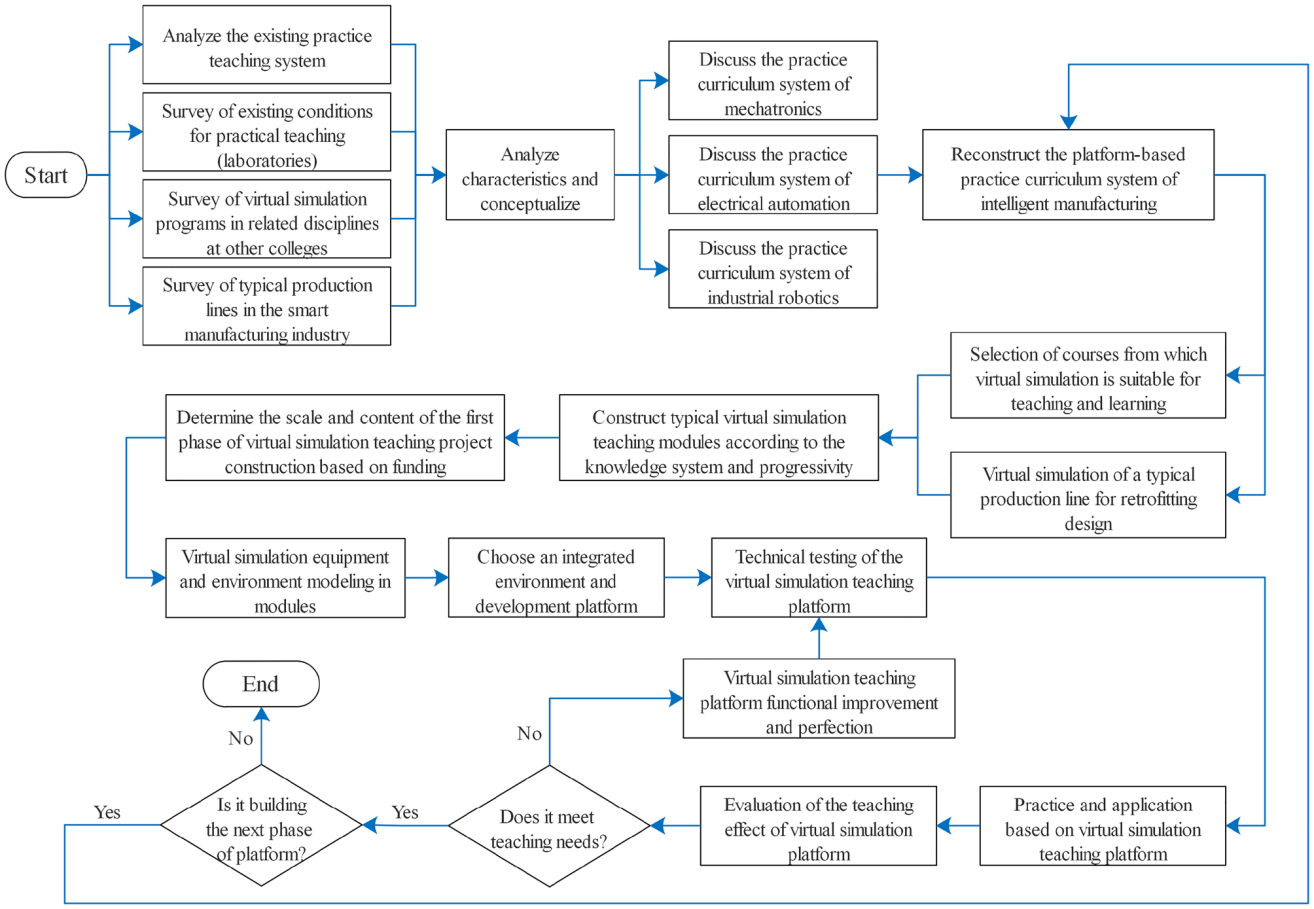
Research flowchart of the study.

At present, engineering training courses in colleges and universities are still in the state of "teaching-based, teacher-oriented", teachers and experimental equipment resources are tight, and it is difficult for students to enjoy "fair" teaching resources. And virtual simulation is the use of computer-generated virtual systems to mimic another real system, including three-dimensional spatial environment of human feelings (vision, hearing, touch) and real-time interactive response fidelity, so as to realize the interaction between the virtual world and the human being, reflecting the real world. China's Ministry of Education is also actively advocating the construction of virtual simulation training environments in colleges and universities, and has issued guidelines for the construction of model virtual simulation training bases. It puts forward the requirements for the construction of virtual simulation training environment, which should be based on the production environment and production equipment of advanced industry enterprises, absorbing new ideas, new technologies, new techniques, new norms and new standards, and constructing a virtual simulation training environment that is docked with the actual vocational situation. In the face of the dual background of the state vigorously calling on local universities to accelerate the construction of virtual simulation training bases and the urgent need to solve the current shortage of practical training equipment for manufacturing majors in engineering colleges and universities. we take the mechatronics technology, electrical automation technology, and industrial robotics technology majors under the equipment manufacturing catalog published by the Chinese Ministry of Education as an example, and propose a set of virtual simulation laboratory construction program to meet their daily virtual simulation teaching needs for the shared course system involved in the above three majors. The program covers the virtual simulation training courses such as electrician and electronic technology, electrical control and PLC, hydraulic and pneumatic technology, industrial robot assembly and debugging, industrial robot on-site programming, touch-screen human–machine interface technology, etc., and takes the first, second, and third year university students of the above three majors as the target service population. Construction of this virtual simulation teaching platform began in 2021 and was completed in 2023, with a total cost of approximately $700,000 USD. The overall flow of this research work, is shown in Fig. 2.

**Figure 3:**
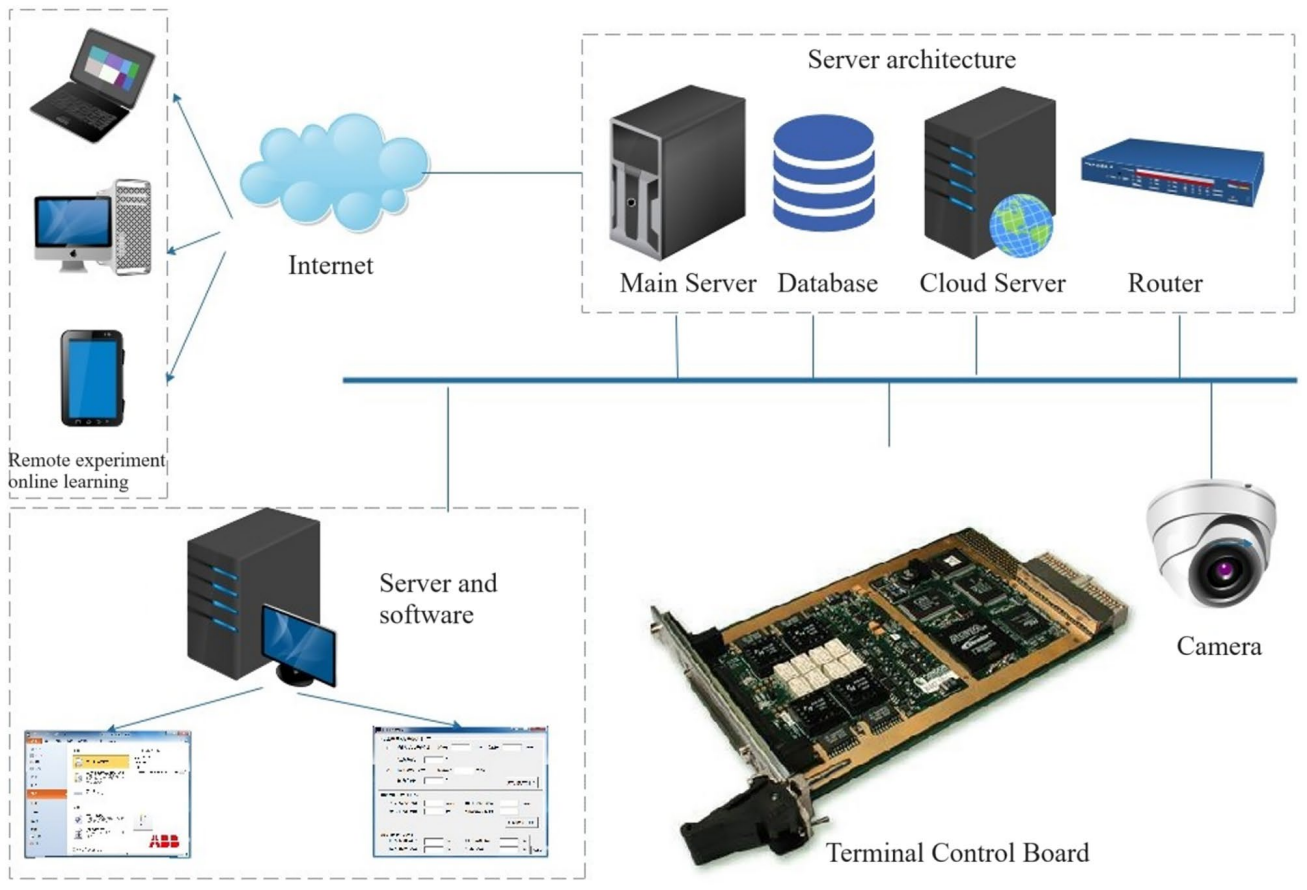
Architecture of the "cloud training" platform.

Accordingly, this study innovates the engineering training teaching mode of engineering majors, takes intelligent manufacturing majors as an example, integrates the practical training equipment, cloud platform, cloud side-end technical means, and utilizes the cloud technology to realize the functions of the practical training equipment such as inbound communication, data transmission, cloud computing, remote operation and maintenance, and so on. Focusing on the shortcomings of the traditional engineering training mode, continuously integrating virtual simulation technology in practical teaching, designing a virtual simulation teaching platform with modern engineering training characteristics. Adopting the idea of "Internet of Everything", realizing the cross-border integration of hardware and software, and realizing the integration across time and space by means of cloud platform. Students can remotely operate on-campus practical training equipment through the digital campus, observe the effect of practical training in real time, and experiment on "cloud", as shown in Fig. 3.

In terms of technological innovation in the design of virtual simulation teaching platform, this paper makes the following contributions: (1) In order to overcome the virtual simulation teaching method of "virtual" but not "real", put forward a combination of virtual and real experimental hardware and software arrangement, the virtual machine and experimental hardware connected to realize the virtual terminal (software) and hardware terminal (equipment) of the two-way interaction control function; (2) In order to break the spatial limitation of the learners, the industrial Internet intelligent transmission terminal equipment (Flexem Box) is used to transmit the experimental commands, programs, and other data of the learners who are outside the laboratory to the industrial equipment in the remote laboratory to carry out the experimental projects of the electrical and electronic class as in the module of section II. Equally, Flexem Box transmits the data from relevant equipment, instruments and video surveillance in the lab back to the cloud data center, helping learners to achieve remote data monitoring, equipment diagnosis and other purposes. Flexem Box device is based on ARM CORTEX A8 processor core, with 3 Ethernet ports, 3 serial communication ports, with WIFI/GPRS/4G and other wireless devices interconnection capabilities. Flexem Box serves as a data bridge between on-site experimental equipment and the learner's client, opening the way to do hardware experiments remotely, realizing that learners can complete electrical and electronic routine experiments at home, and the results of the experiments are real and visible (observing the relevant data in the lab and the execution of the institution through the remote camera). This is a significant innovation of the virtual simulation teaching platform technology program proposed in this study, and is also a breakthrough in the integration technology of the virtual simulation teaching platform of intelligent manufacturing class, which provides an effective attempt to solve the limitation that the hardware experiments can only be carried out offline for a long time; (3) The virtual simulation teaching platform developed for intelligent manufacturing specialties promotes the balanced distribution of engineering practical teaching resources, overcomes the limitations of traditional practical teaching limited by the number of sets of equipment, and provides learners with more operating opportunities and trial and error possibilities. Additionally, many practical contents of intelligent manufacturing specialties have certain safety risks, such as electricians and machine tool operations, while the virtual simulation platform supports design, testing and simulation operations, which helps to improve the practical operation skills of learners and allow them to expand more practical knowledge in a safe environment; (4) Virtual simulation teaching platform through virtual reality technology, can simulate a variety of real or difficult to actually operate the teaching scene, such as electrical fault debugging, personalized integration of the production line, the disassembly of the robot body. This high simulation virtual teaching environment not only enhances the sense of real experience and learning fun when the learner operates, but also allows the learner to intuitively understand and master complex knowledge points, effectively making up for the classroom presentation of many difficult to reproduce, difficult to disassemble the operational content. Therefore, this virtual simulation teaching platform for intelligent manufacturing class has a significant competitive advantage in the teaching of engineering specialties, especially in the vocational education scenario that pays more attention to the cultivation of practical operation ability. Since this platform is designed based on the concept of intelligent manufacturing class professional group, and the teaching design of virtual simulation module is carried out according to the basic platform courses of each similar specialty, it is not only applicable to electromechanical specialties, but also can meet the basic experimental teaching of electronic information specialties. This virtual simulation teaching platform designed based on the course content of the multi-specialty platform has obvious competitive advantages over the virtual simulation platform for a single course in terms of the radiance of the teaching application level and the logic of the complete knowledge system. It will be able to play its own strengths in avoiding repeated investment in construction costs and realizing cross-specialty and cross-school practical teaching resource sharing.

### Electrician electronics and PLC control virtual simulation module

**Figure 4:**
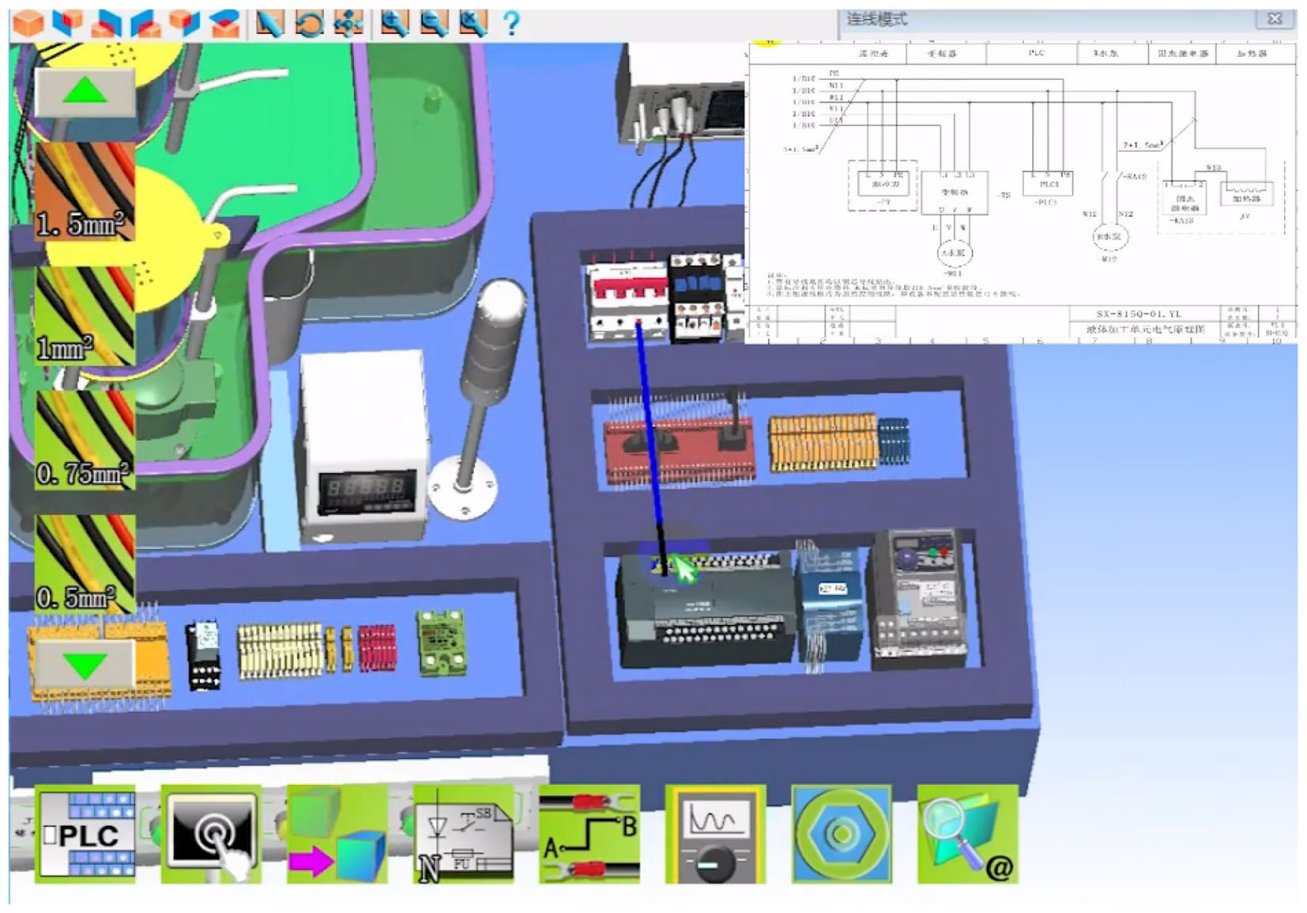
Virtual simulation of electrical wiring.

**Figure 5:**
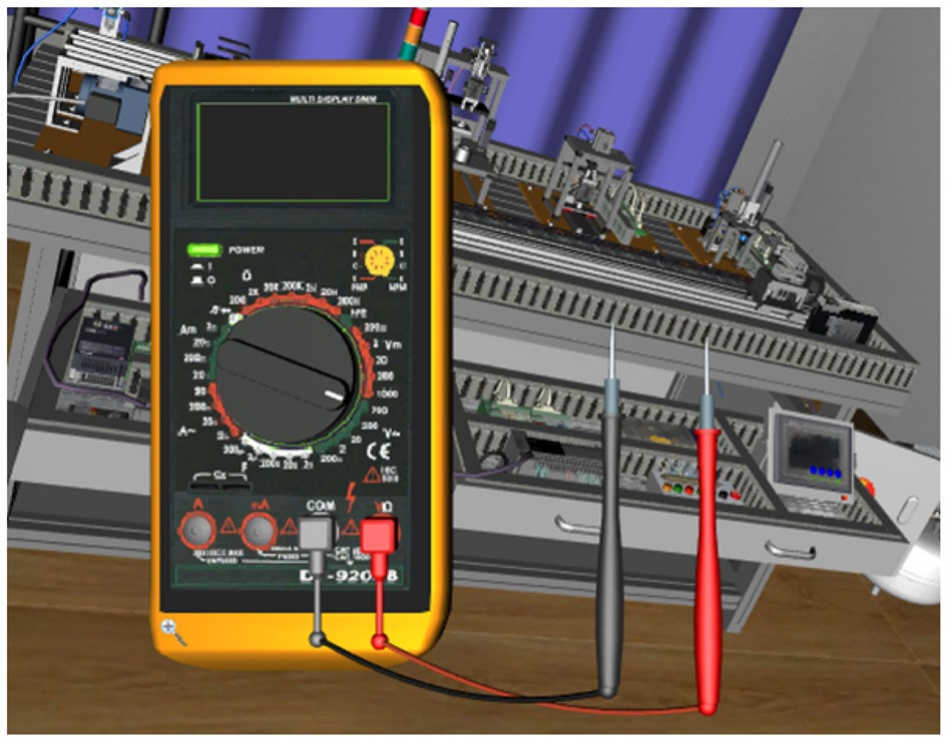
Measurement with virtual multimeter.

**Figure 6:**
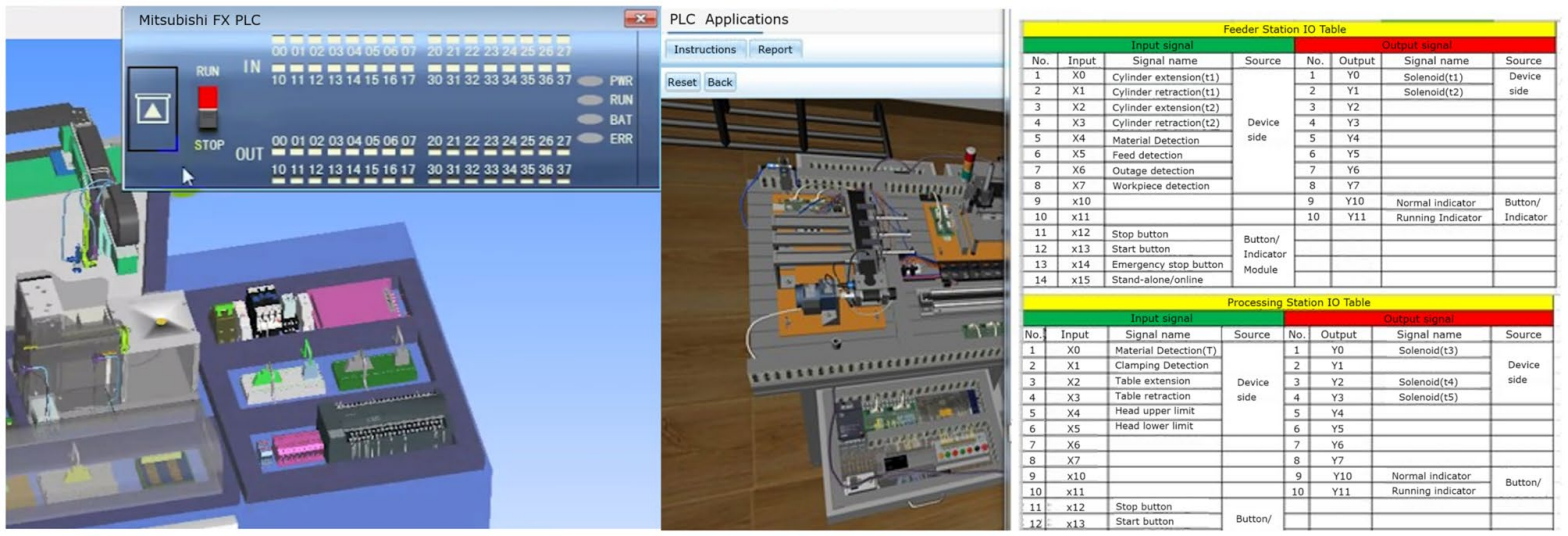
Virtual simulation of I/O Setting Interface in PLC Programming.

The module can carry out conventional electrical components and wiring, stepper motor, DC motor, AC motor control and its programming and communication experiments. Three-dimensional virtual wiring using three-dimensional physical circuits and two-dimensional planar circuits, both in three-dimensional physical circuits can be wired, but also in the two-dimensional planar circuit operation, as shown in Fig. 4. Meanwhile, the module also has the function of circuit logic analysis, which can monitor the current flow and the execution action of electrical components in real time. The virtual multimeter can measure voltage and current in the 3D and 2D scene to view and detect faults in real time, as shown in Fig. 5. PLC can be edited and written in the virtual scene to control the operation of the stepper motor, the external components according to the signal drive to perform the corresponding action, and real-time observation of the operation of the stepper motor and PLC and other components, such as faults will be alarmed in real time and indicate the possible location of the fault, as shown in Fig. 6.

The main function of the module is to meet the learners of the basic electrical control lines and PLC control line installation and adjustment operations, as shown in Fig. 4, the learner according to the electrical schematic diagram provided by the system, in the virtual environment to complete the selection of the corresponding electrical components, installation and wiring operations. And can use the virtual multimeter on the completion of the electrical control line in the corresponding nodes of resistance, voltage, current and other basic physical measurements, simulating the debugging and troubleshooting operations of the electrical line. In addition to the operation of virtual control lines based on basic components such as relays, contactors, travel switches, etc., this module also has the function of realizing the virtual debugging operation of basic control lines using PLC controllers, and the learner can connect the PLC with the built-in virtual control scene equipment through virtual wiring, such as the experiment of the material loading control unit in Fig. 6, and the user can set up PLC's input and output ports and monitor the real-time signals of the relevant ports. The virtual operation of all the functions of this module supports two user interface interaction methods: mouse + keyboard, VR glasses + virtual control handle.

### Virtual-real module of intelligent manufacturing typical production line.

**Figure 7:**
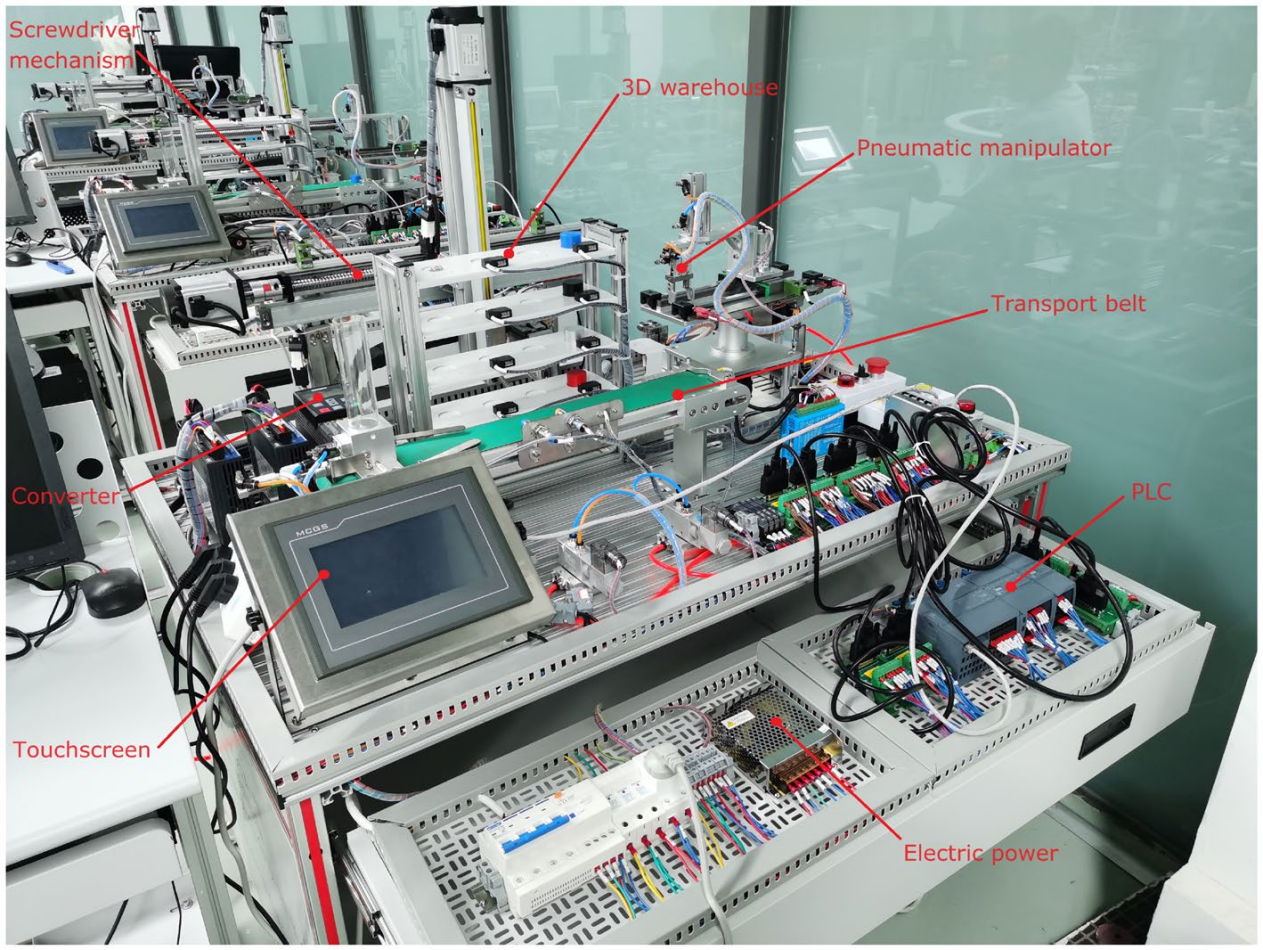
Experimental bench of typical production line.

**Figure 8:**
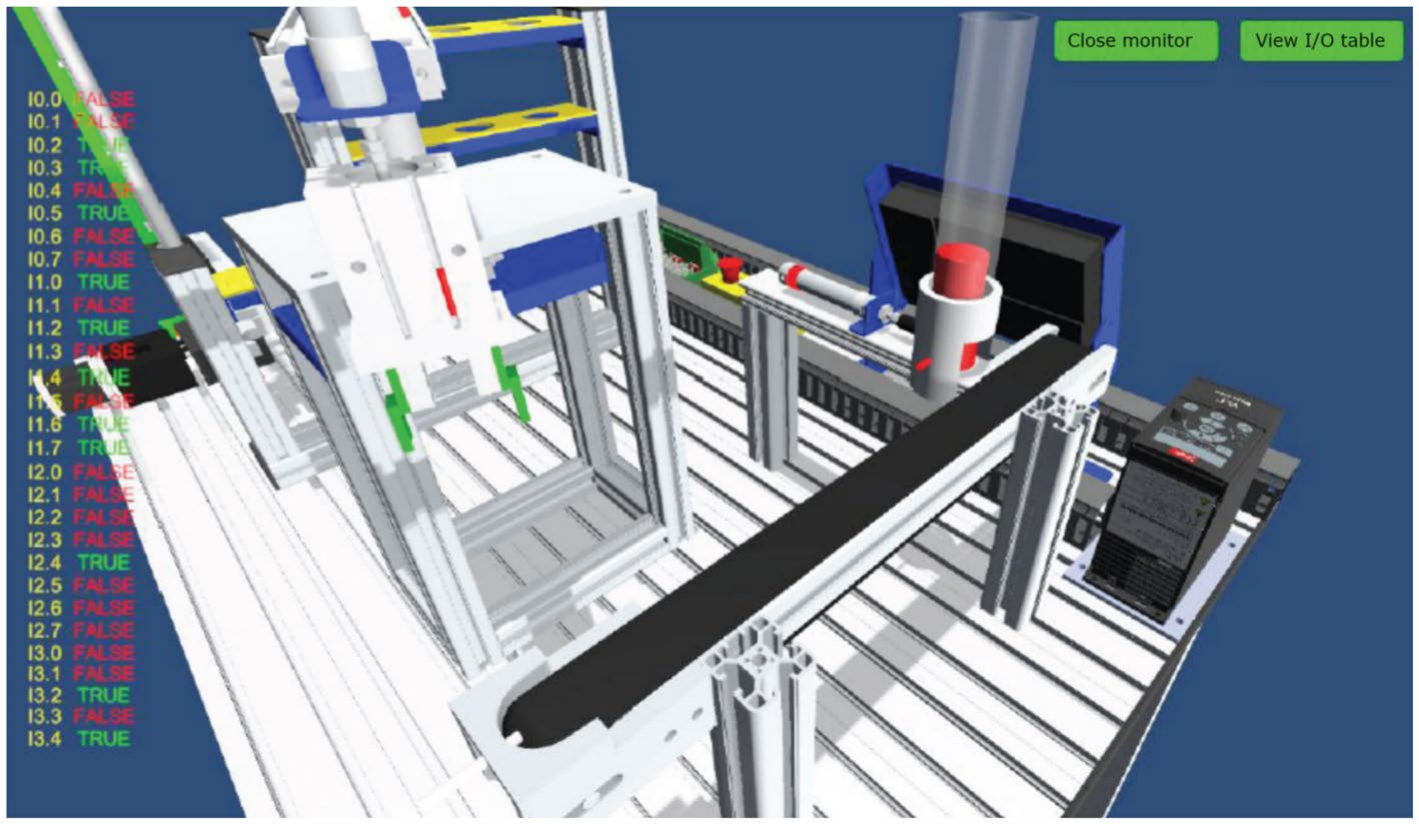
Virtual production line status monitoring (input signals).

**Figure 9:**
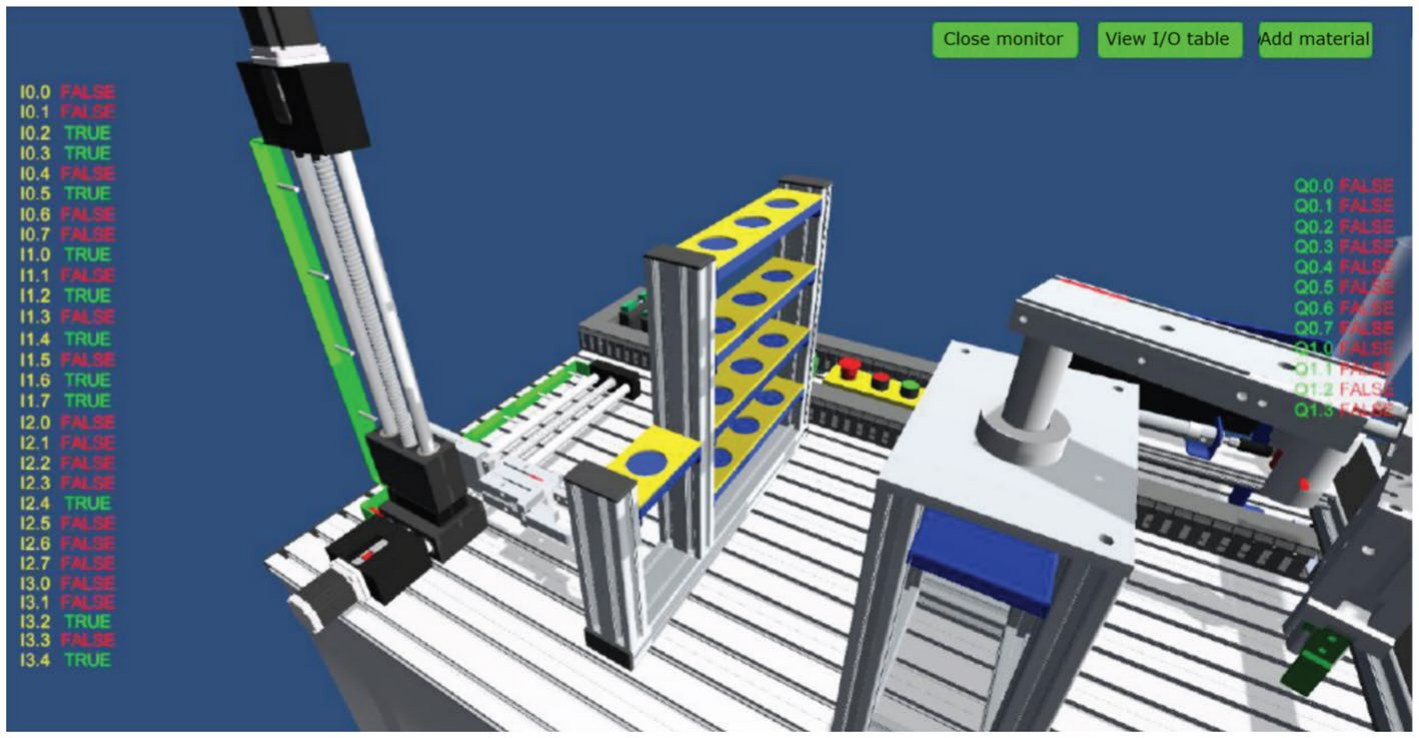
Virtual production line status monitoring (I/O signals).

**Figure 10:**
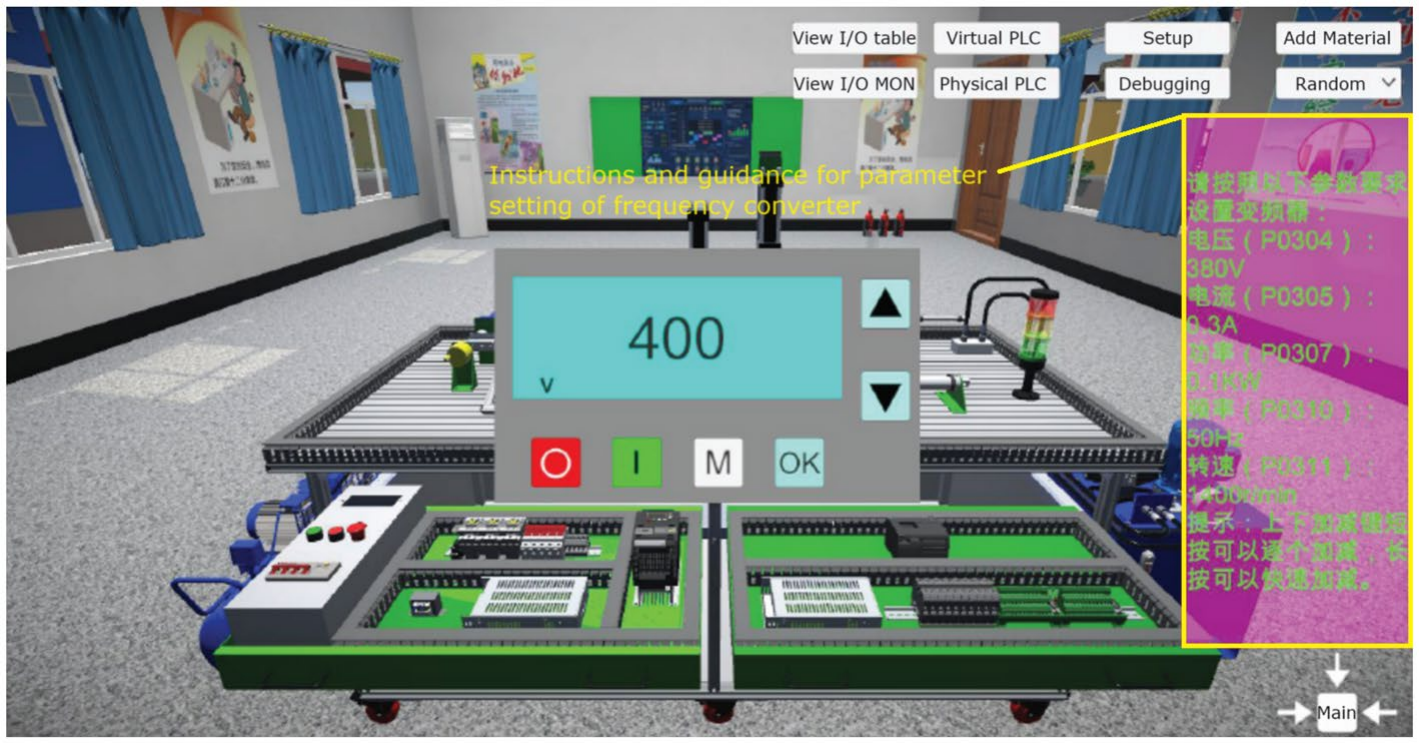
Converter setting interface.

**Figure 11:**
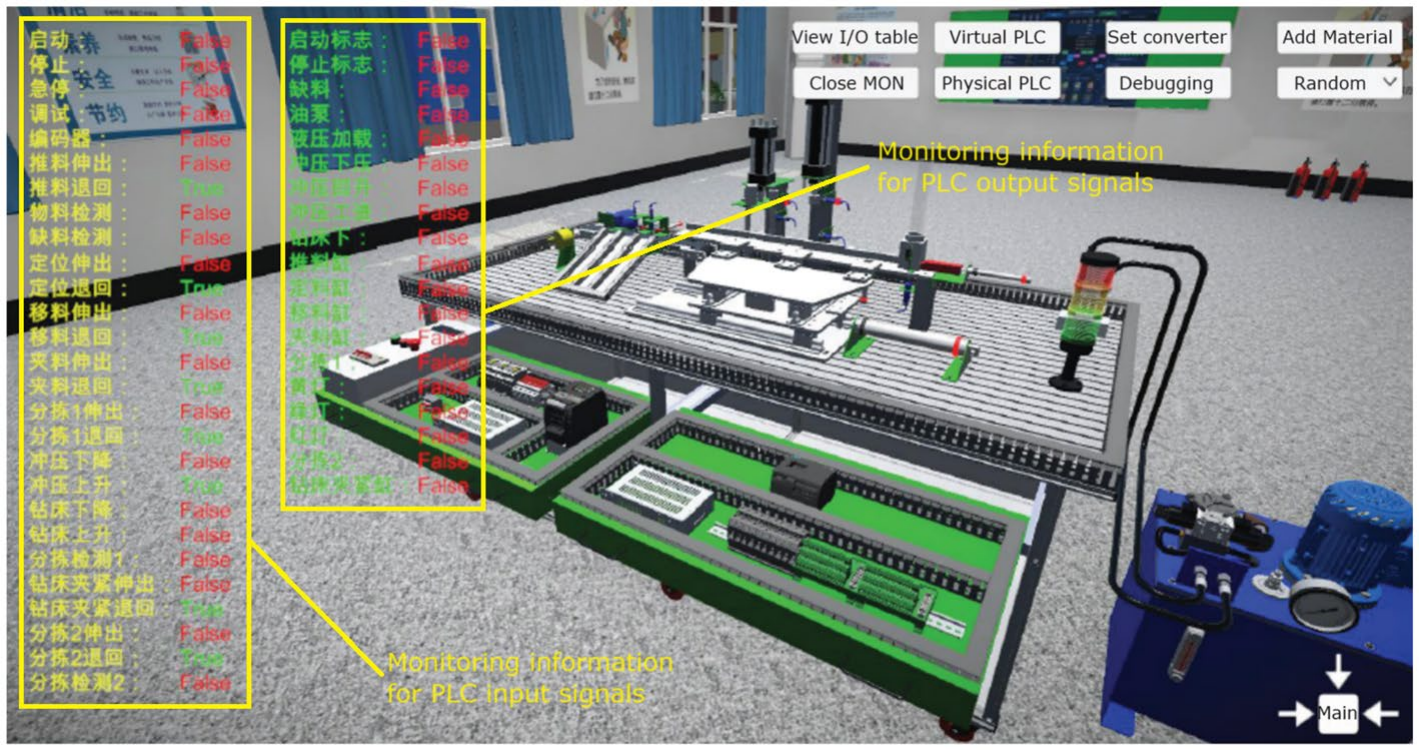
Panoramic view of the virtual production line.

This module integrates mechanical, pneumatic, electrical control, motor drive, sensor detection, PLC and industrial network control technology, and designs a set of virtual-real combination of practical training platform to meet the typical application scenarios of automated production line. The practical training platform consists of loading conveyor, handling robot, stacking robot, three-dimensional warehouse and other mechanical devices, can meet the automated production line, mechanical and electrical integration system design and other courses of practical training and teaching needs. The mechanical structure and control of the practical training table adopt unified standard interface, which has high compatibility and expandability. It can be used for teaching the installation and debugging of mechanical components, installation and debugging of pneumatic systems, installation of electrical control circuits and PLC programming, etc. It is suitable for single skill training and comprehensive project training for intelligent manufacturing majors, as shown in Figs. 7, 8, 9, 10 and 11.

The internal scenes in the simulation module, such as loading conveyor lines, handling manipulators, stacking manipulators, three-dimensional warehouse, etc., are built according to the real parts of the proportional size, and all the equipment are physical simulation of the operation mode corresponding to the real parts. The production line is divided into "physical control mode", "virtual control mode", "view status monitoring" and other operating modes, and realizes the virtual environment control virtual objects, real environment control virtual objects, real environment control physical objects and virtual environment control physical objects, virtual and real interoperability, cross-linkage operation.

The main function of this module is to integrate the simulation of units such as conveyor belts, manipulators, and three-dimensional warehouses that are commonly involved in typical automated production lines. The system built-in material conveyor, workpiece clamping and handling robot, screw drive module, three-dimensional warehouse, air compressor, PLC controller, frequency converter, electrical control module, equipment racks and other models are arranged into the virtual environment by learners. The virtual wiring function is used to connect the lines of each unit, the PLC controller is used to assign the input and output addresses of the controlled objects of each unit, and finally the linkage and debugging of the whole production line is controlled through PLC programming. The virtual operation of all the functions of this module only supports the user interface interaction mode of mouse + keyboard, and the human–computer interaction mode of VR headset + digital gloves will be introduced in the future.

### Dual-axis collaborative robotics workstation module

**Figure 12:**
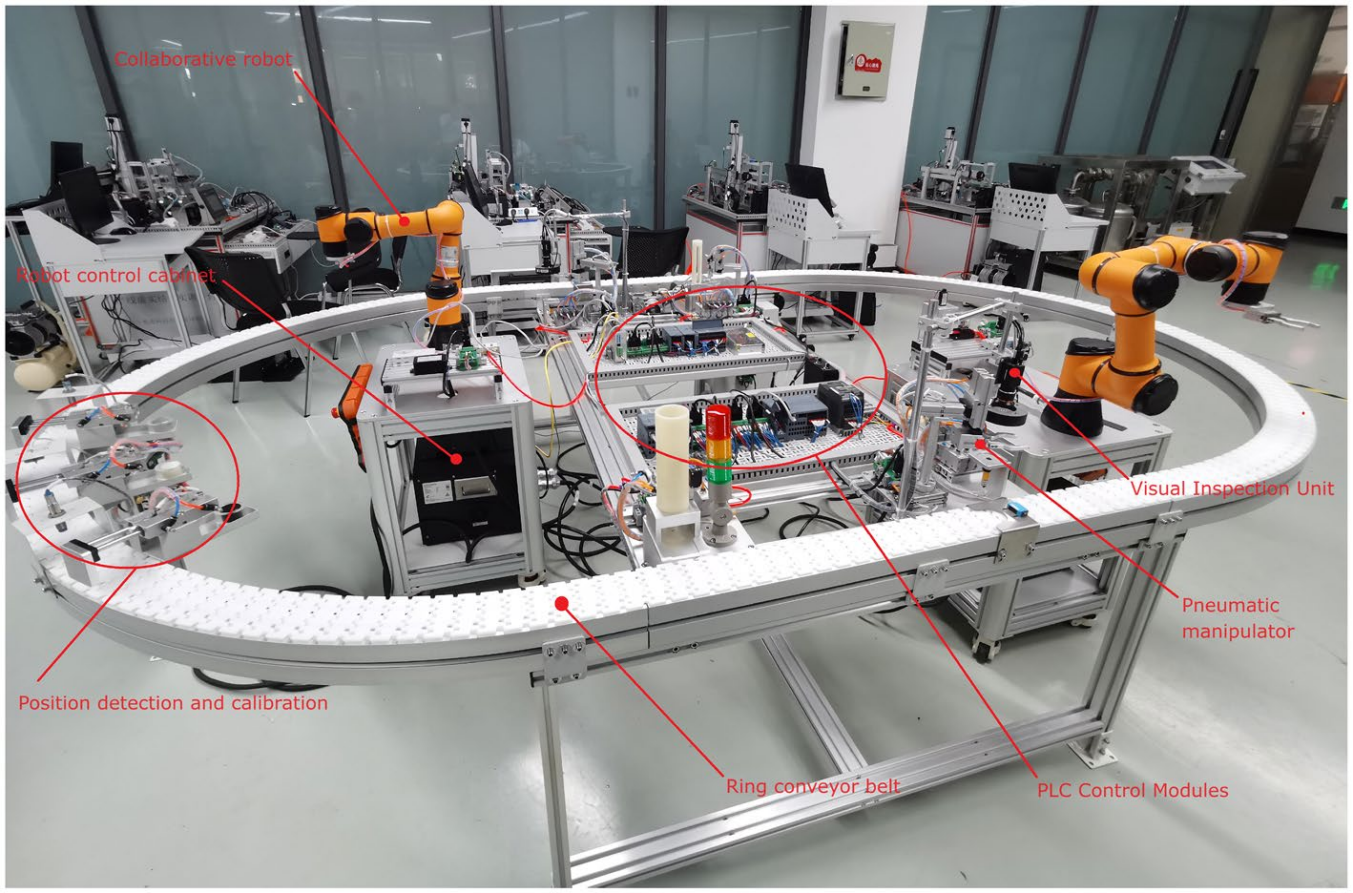
Real view of two-axis collaborative robot workstation.

After completing the above basic unit knowledge modules, learners can commission the units on-line to learn control, programming, assembly and commissioning techniques for complex systems. Therefore, we designed a two-axis collaborative workstation for an intelligent manufacturing line based on digital twin technology, which consists of six execution units for loading, machining, packaging, testing, and sorting, including a ring production line system, a vision system, with two 6-axis collaborative robots. The components on this workstation are modularized and can be used to teach the installation and debugging of electromechanical equipment, the installation and debugging of ring automatic control systems, and the installation and debugging of industrial network control systems, as shown in Fig. 12. This module is a physical workstation, only one set of which is currently laid out for funding reasons, and will be used in conjunction with the digital twin simulation module in Sect. "digital twin simulation module" in teaching.

### Digital twin simulation module

**Figure 13:**
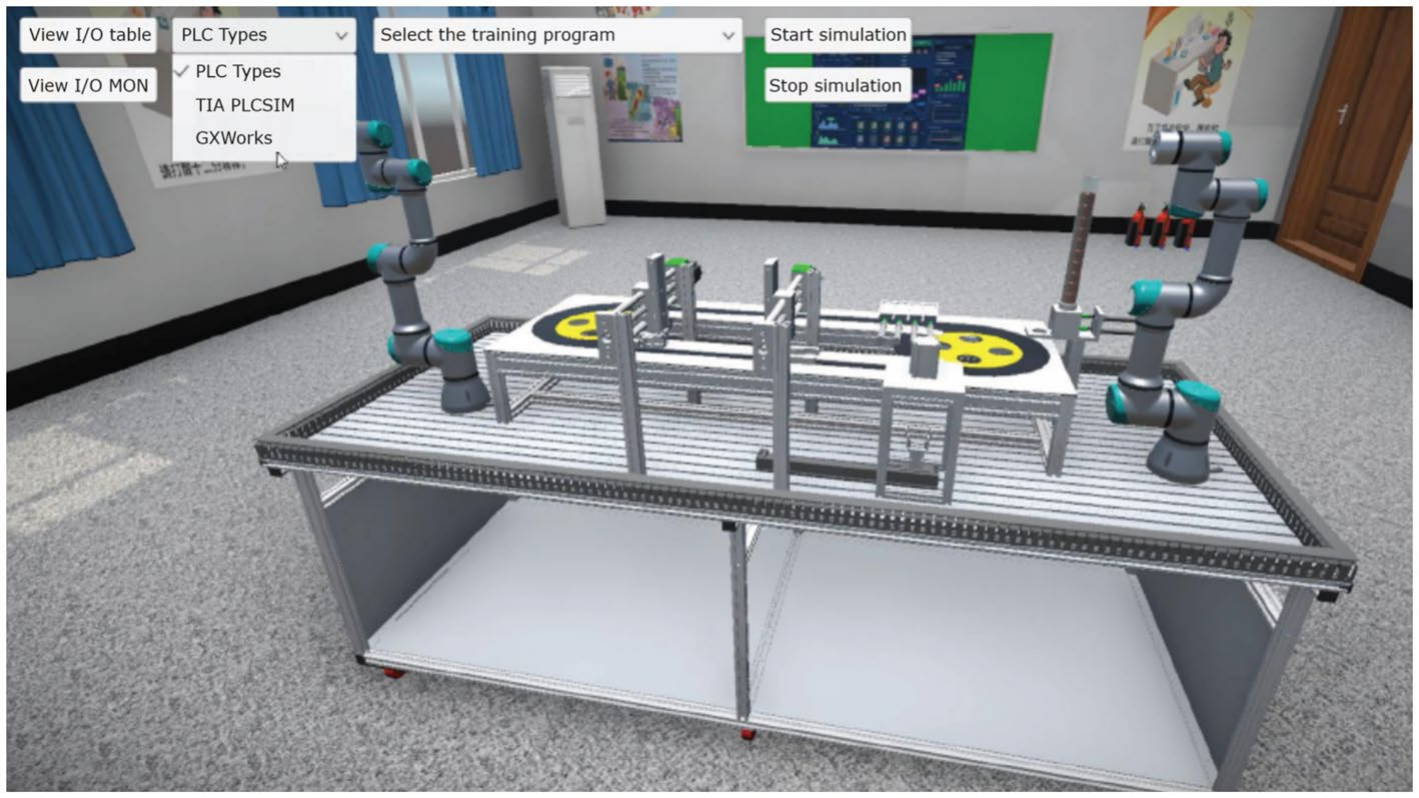
Panoramic view of the digital twin simulation module.

Due to the expensive cost of the dual-axis collaborative workstation equipment, in order to meet the needs of more students to use it and save the cost, we designed a simulation module based on digital twin technology for teaching dual-axis collaborative workstation in a digital environment. The module uses UNITY engine, 3DMAX modeling, to build a VR three-dimensional engine, editing and development platform, which can be used to browse the scene through VR, support spatial positioning, and can manipulate the handle to operate in the virtual scene of the software system, and watch the structure of the equipment and operate some specific experimental steps through the form of VR, as shown in Fig. 13.

**Figure 14:**
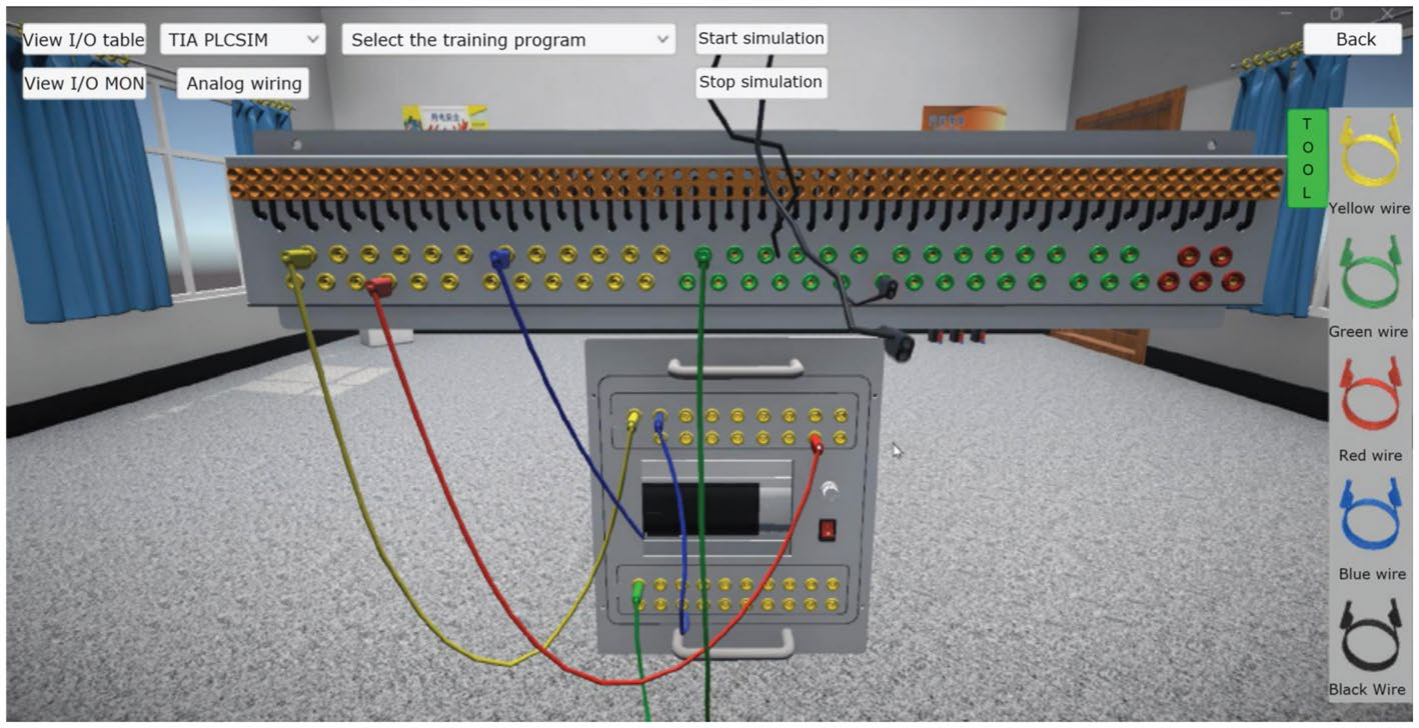
Simulation of PLC communication in digital twin.

The module can be connected to external real PLC through the built-in communication driver, and can also be connected and communicated with virtual PLC, such as PLCsim, as shown in Fig. 14. The module integrates a robot model library, covering most robot brands, and supports the import of 3D model data and the addition of non-standard model components to the component library. Students can build a virtual production line through the mouse drag-and-drop operation, and give it motion parameters and control instructions, etc., can better simulate the simulation of real equipment commissioning state.

This module has the functionality of the module in Sect. "Virtual-real module of intelligent manufacturing typical production line" with the addition of a 6-degree-of-freedom virtual collaborative robot and visual inspection. According to the site and to facilitate teaching, the linear conveyor belt is changed into a ring, and two collaborative robots are used to realize the transfer of workpieces between work processes. The collaborative work operation function of the robots is emphasized to meet the learners' training needs for online programming and operation of robots. At present, the virtual operation of all the functions of this module only supports the user interface interaction mode of mouse + keyboard.

### Virtual disassembly module for industrial robots

As a typical execution unit in the intelligent manufacturing industry, industrial robots have been widely used in various industries in the field of equipment manufacturing. Therefore, knowledge about the internal mechanical structure of industrial robots and their electrical control systems is also an essential professional foundation for students majoring in intelligent manufacturing. However, due to the high price of the industrial robot body, if frequent repetitive disassembly of the industrial robot body is easy to reduce the positioning accuracy of the robot and accelerate the obsolescence of the equipment, it is not able to meet the long-term repetitive daily teaching needs. Therefore, we use MAYA modeling, Unity3D running platform, design industrial robot virtual disassembly simulation module, based on dynamic simulation, combined with 3D virtual reality technology and SteamVR asynchronous projection technology to achieve the virtual disassembly of industrial robots, so that the operator is like an immersive roaming and interactive operation in the scene.

**Figure 15:**
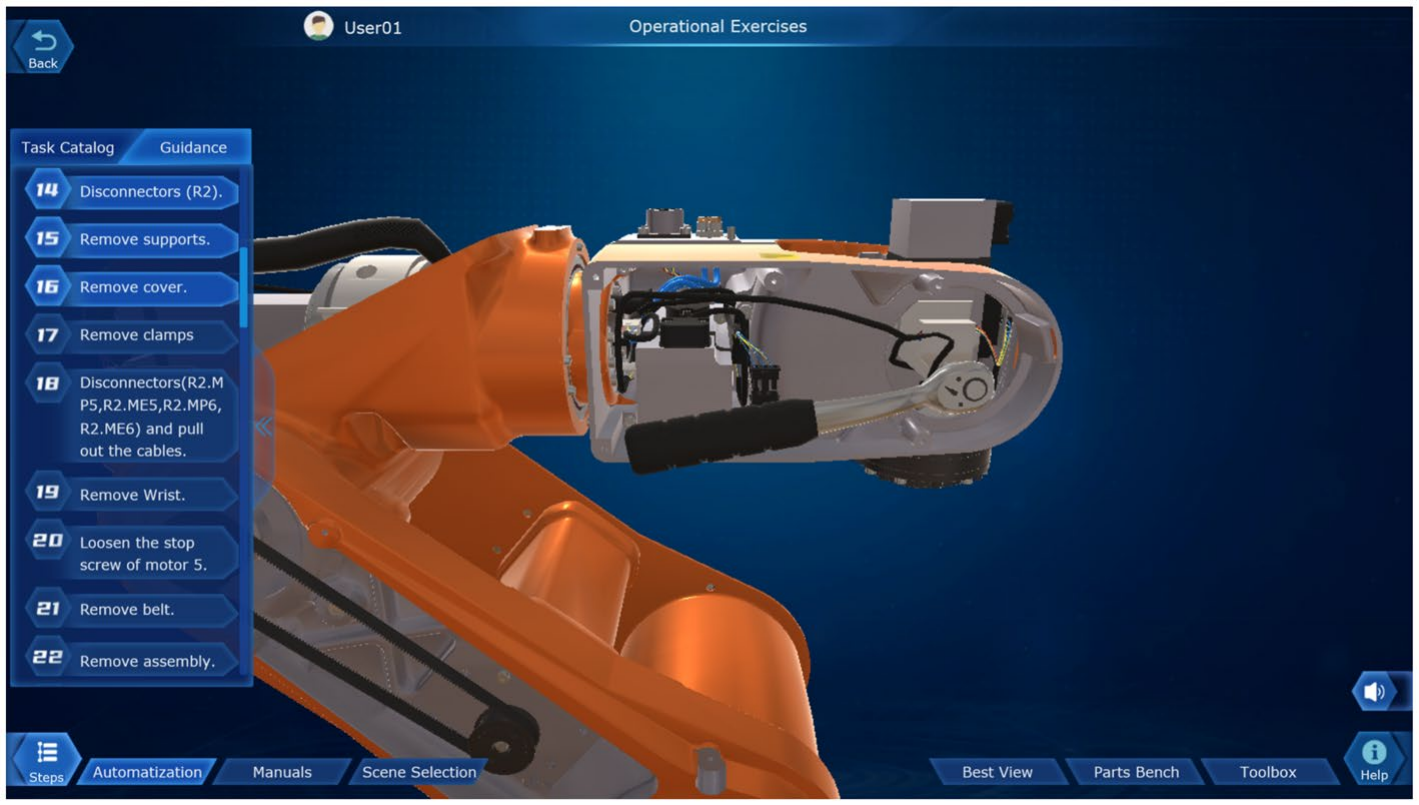
Virtual disassembly of industrial robot body.

**Figure 16:**
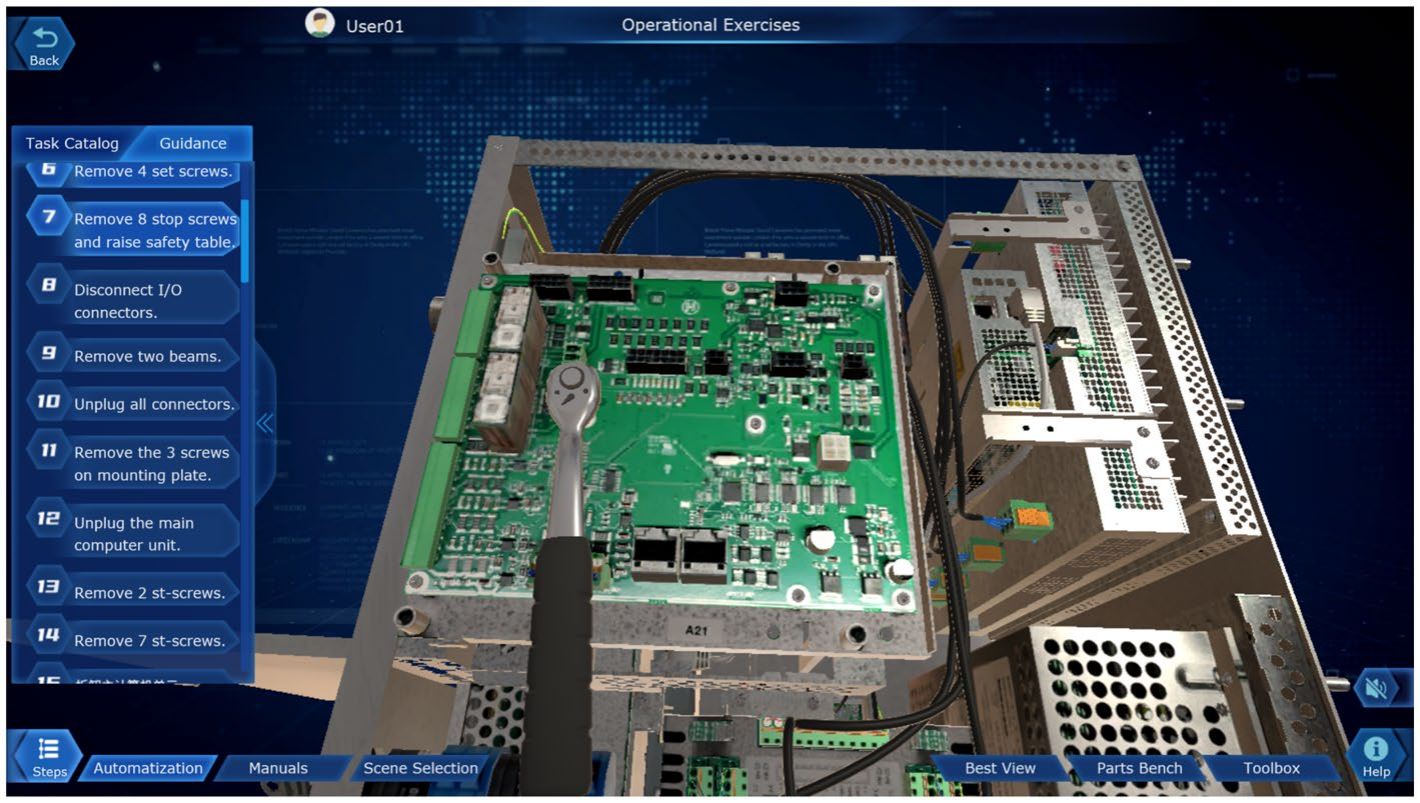
Virtual disassembly of the electrical control cabinet of industrial robot.

Take a typical industrial robot ABB IRB 120 arranged on campus as an object, the core parts of the robot body (upper and lower arms, wrist side cover and housing, connectors, timing belt, harness bracket, reduction gearbox assembly, electrode assembly, joint assembly, base, etc.) and the key parts of the electrical control cabinet (cover plate, fan cover, power distribution unit, computer unit, circuit boards, power supply connector, drive unit, cables, etc.) are mapped and modeled. This module covers the three major teaching demonstration functions of virtual disassembly, installation, and maintenance, and uses multiple small tasks for training in the disassembly and installation of each axis and complete robot. Among them, it contains bolt disassembly sequence, bolt installation torque, parts lubrication, etc., as shown in Fig. 15. Additionally, through the interactive operation of the handle and the scene, the robot electrical control cabinet can be disassembled and assembled, and the electrical components can be arbitrarily rotated, translated, enlarged, and reduced, and simulated disassembly, installation, and maintenance can be carried out in accordance with the technical specifications for maintenance, as shown in Fig. 16. The virtual disassembly operation of this module supports two user interface interaction modes: mouse + keyboard, VR glasses + virtual control handle.

### Virtual simulation module for magnetic yoke shaft flexible production line

**Figure 17:**
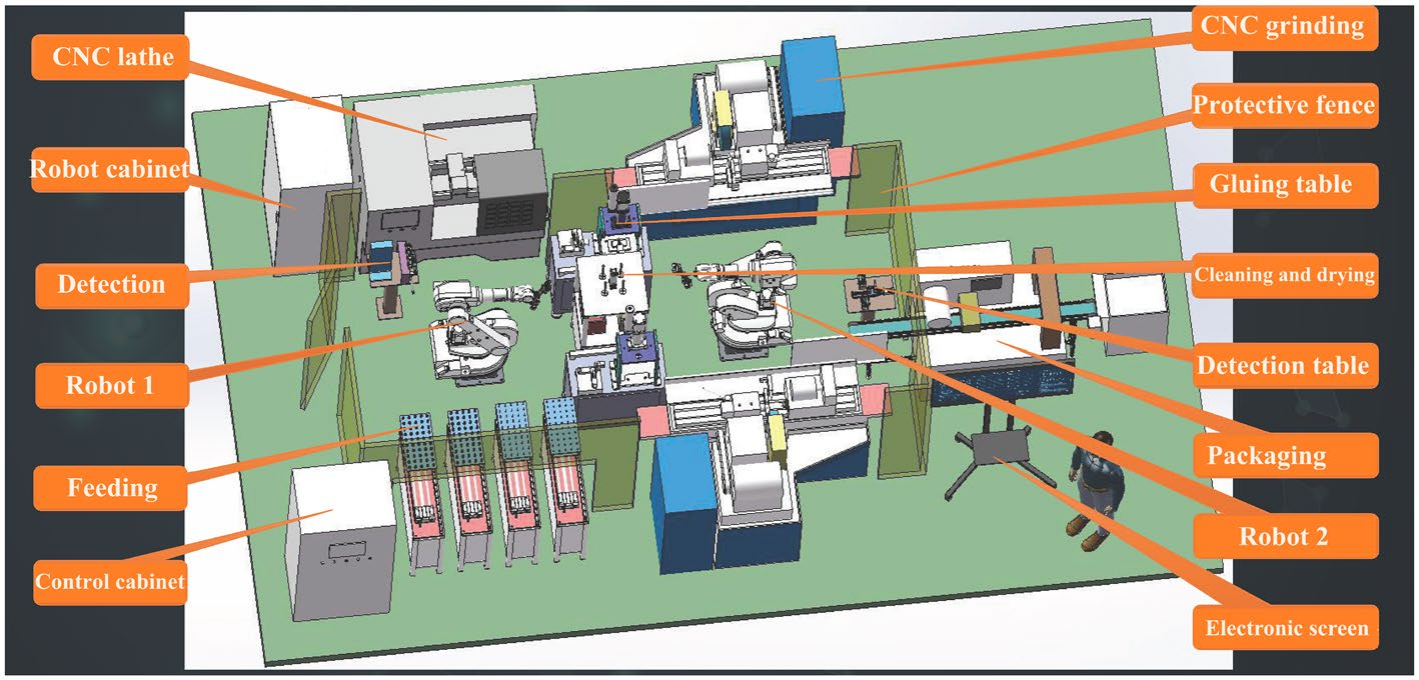
Virtual simulation module for flexible production line of magnet yoke shafts.

On the basis of the above virtual simulation module, an enterprise product production line is introduced through the form of school-enterprise cooperation, and the virtual simulation teaching is carried out with real product cases, so as to make job connection for students' employment in advance and further improve the social adaptability of this virtual simulation teaching platform. Take the yoke shaft in the miniature special motor as the production object, a virtual simulation automated production line is established through three-dimensional simulation technology, and the module contains a heat treatment equipment, two CNC lathes, a CNC grinder, and two robotic arms. The machining process includes: yoke turning, dimensional inspection, yoke shaft finishing, yoke shaft grinding, cleaning and drying, dimensional inspection, and packaging of finished products, as shown in Fig. 17. Simultaneously, the Internet of Things sensor data of the real production line MES system is associated with the three-dimensional scene and equipment in the virtual simulation module, and real-time data display is carried out on the virtual simulation model to realize the three-dimensional visualization display of dynamic data, and real-time update of the equipment stations in the three-dimensional scene, sensors, and the operation status of monitoring equipment. The module supports learners to build flexible production lines by themselves through typical equipment libraries (CNC lathes, CNC milling machines, CNC grinding machines, heat treatment machines, industrial robots, conveyor belts and other organizations), and also supports models in stl, obj and other formats to be imported into equipment libraries. Learners can conduct virtual simulation of typical production steps involved in the machining process of magnetic yoke shafts, such as CNC roughing, fine turning, fine grinding, workpiece clamping and transfer, cleaning and packaging. The virtual drive of CNC machine tools and industrial robots moving trajectories is performed by inputting PLC control commands and NC machining codes, in which the NC codes are only used for start-stop simulation of CNC machine tools, and the effect of machining parameter settings and G-command operation is carried out in another specialized CNC simulation software. Students can use this module to complete the production line equipment construction, related equipment knowledge learning, knowledge assessment and other functions. It can meet the teaching needs of comprehensively applying relevant knowledge and skills to design and debug a small flexible production line relatively independently.

The main functions of this module include the reasonable layout of the flexible production line unit equipment, production line construction steps of the virtual simulation and the simulation of the machines and robots work beat control training, focusing on the communication between multiple devices and process coordination simulation operation. At present, the virtual operation training of this module is carried out in the professional computer room, and learners can carry out virtual simulation operations such as layout, PLC programming and importing for each equipment in the production line through the interaction of mouse + keyboard.

## Applications and discussion

In order to continuously improve the application level of new-generation information technology such as virtual reality and artificial intelligence in practical training teaching, and deeply integrate information technology and practical training facilities, this work constructs a virtual simulation training teaching platform with perception, immersion, interactivity, conceptualization, and intelligence, and builds a set of virtual simulation training system with virtual and real combination, and configures the corresponding virtual simulation training equipment, which effectively solves the difficulties in the process of practical training teaching.

We innovate the traditional practical training teaching methods by adopting the hybrid practical training teaching method, which is mainly based on virtual simulation teaching, double verification of virtual and real combination, and the combination of online and offline linkage test and Q&A. The teaching process is described as follows: the practice place is divided into three kinds of on-campus pure equipment training room, pure virtual simulation training room (computer), virtual and real combination of training room (computer & equipment). Among them, the pure equipment training room such as the above mentioned flexible production line (hardware) training room, the production line from the enterprise special motor shaft of the real production line, due to the equipment is only a set, so only for trainee; Pure virtual simulation training room (computer) as mentioned above section II of the industrial robot virtual disassembly and assembly and flexible production line virtual simulation, the training room consists of computers and secondary development of the corresponding software, the software is installed in the cloud server on campus, the local computer room as a mirror of the software, students are required to log in and use the software through the unified server platform on campus, which supports simultaneous operation of 50 nodes; The combination of virtual and real training room such as section II mentioned above, which arranges the training equipment and virtual simulation software in the same place, and the students first utilize the simulation software in the computer to conduct virtual simulation on the teaching content. For example: the use of handling robot to achieve the handling of specified items into the warehouse experiment, students need to use the computer to set up the point of the robot's trajectory, and through the PLC to write a simple program to control the robot to the items of the grasping, moving, and warehousing, the virtual simulation run in the software environment. On this basis, students connect and communicate the computer with the real PLC through the data line, and write the program after the simulation is verified to be correct into the real PLC, and drive the real manipulator to carry the specified items and put them into the warehouse for debugging, which is a simple teaching case of the virtual-real combination training. All the computers in the virtual simulation training room mentioned above use Intel processors, Windows 10 operating system, 16.00 GB of RAM, processor Intel Core i7-10700, 8 cores and 16 threads, main frequency 2.90 GHz, and graphics card NVIDIA GeForce GTX 1650, simulation software based on customized training equipment for secondary development, the PLC library in the simulation system supports Siemens S7-200, S7-1200, S7-300, S7-400, S7-1500 series and Mitsubishi FR-FX2N, FR-FX3U, FR-FX2NC series, etc., and it does not support the customization of adding other domestic models of PLC, and the hardware PLC adopts the Siemens 1214C and Mitsubishi FR-FX3U series, and it supports the ladder and SFC as the programming languages, and other hardware using standard parts integration.

Students gave their informed consent to the processing of their data. The study was approved by the Yiwu Industrial and Commercial College and was in accordance with the principles of the Declaration of Helsinki.

### Teaching application of simulation platform

The innovation of virtual simulation teaching platform must be applied to teaching practice as an important hand and impetus to promote the development of online education, alleviate the current tense situation of practical teaching equipment, and improve students' hands-on opportunities. As consequence, we carried out the practice based on the virtual simulation teaching platform for the intelligent manufacturing majors (including electrical automation technology, mechatronics technology, and industrial robotics technology) in our college. Two parallel classes (Class A and Class B) of different grades of each specialty are randomly selected as practice objects, in which Class A (Automation Class A, Mechatronics Class A, Robotics Class A) of all the specialties as the first group of learning samples, using the virtual simulation teaching platform established in this paper to carry out the daily teaching and learning implementation, and Class B (Automation Class B, Mechatronics Class B, Robotics Class B) as the second group of learning samples, maintaining the original mode of teaching and learning, and adopting the grouping of batch multi-persons to share the same set of real equipment to carry out the teaching and training, so as to carry out the data analysis on the mastery of the various core knowledge points.

In order to reflect the differences between the two sets of teaching modes more comprehensively and objectively, we also designed a test bank for different levels of mastery of the core knowledge points of different specialties (including theoretical and practical knowledge). Industrial robotics technology, for example, the test bank covers the basics of industrial robotics (theory), common electrical wiring installation and commissioning (practice), common PLC control line installation and commissioning (practice), industrial robotics system offline programming and simulation (practice), industrial robot assembly and overhaul (practice), industrial robotics application system integration (practice) and other 6 knowledge modules, each of which consists of 10 theoretical or practical questions with 10 points each. The composition of the questions for the industrial robot application system integration module, as shown in Table 2. In addition, learners' subjective feelings were further investigated through the student experience of using the questionnaire feedback form, as shown in Table 3, and the four options for questions 4–15 in the Table 3 are scored 5, 3, 2 and 1.

**Table 2:** Sample questions for the knowledge module on system integration for industrial robot applications.

Nos.	Name of modules	Test questions	Marks
1	Module 6	Servo motor drive configuration and application experiment	10
2	Module 6	Programming control virtual real-time simulation system experiment	10
3	Module 6	Electromechanical gas–liquid integration simulation experiment	10
4	Module 6	Programming and debugging of fieldbus communication technology	10
5	Module 6	Installation and debugging of machining unit	10
6	Module 6	Installation and debugging of pneumatic manipulator unit	10
7	Module 6	Mechanical installation and adjustment of assembly unit	10
8	Module 6	3D industrial camera detection and application experiments	10
9	Module 6	PLC and robot arm communication control experiment	10
10	Module 6	Cooperative operation of robot and assembly line	10

**Table 3:** Learners' experience questionnaire.

Questionnaire on students' mastery of the specialized knowledge they have learned (Intelligent Manufacturing)		
No	Issues	Options
1	What is your gender?	○ Male ○ Female
2	What is your major?	○ Mechatronics ○ Electrical automation ○ Industrial robot
3	What is your grade?	○ Freshman ○ Sophomore ○ Junior ○ Senior
4	Are you very interested in your field of study?	○ Perfectly match ○ Generally match ○ Not sure ○ Not match
5	Do you think that your field of study will be of great help in your future employment?	○Perfectly match ○ Generally match ○ Not sure ○ Not match
6	How often do you read books or papers related to your field of study?	○Often ○ Occasionally ○ Infrequently ○ Never
7	How is your status in class?	○Serious ○ Sloppy ○ On my phone ○ Sleeping
8	At the end of the day, how well did you grasp the knowledge of the lessons you learned that day?	○Completely mastered ○ Mostly mastered ○ About half ○ Not at all
9	Do you preview and review what you have learned in advance?	○Perfectly match ○ Generally match ○ Not sure ○ Not match
10	What is your favorite teaching style?	○Virtual and real ○ Physical manipulation ○ Virtual simulation ○ Knowledge teaching
11	Can you disassemble and recover industrial robot bodies independently?	○Perfectly match ○ Generally match ○ Not sure ○ Not match
12	Can you independently connect between the robot and the PLC?	○Perfectly match ○ Generally match ○ Not sure ○ Not match
13	Can you independently install and commission a simple manufacturing unit?	○Perfectly match ○ Generally match ○ Not sure ○ Not match
14	Can you independently build and co-commission a simple automatic production line?	○Perfectly match ○ Generally match ○ Not sure ○ Not match
15	Do you think you will be able to perform the basic tasks of a relevant position in the field of smart manufacturing after graduation?	○Perfectly match ○ Generally match ○ Not sure ○ Not match

### Discussion and evaluation of teaching effectiveness

**Figure 18:**
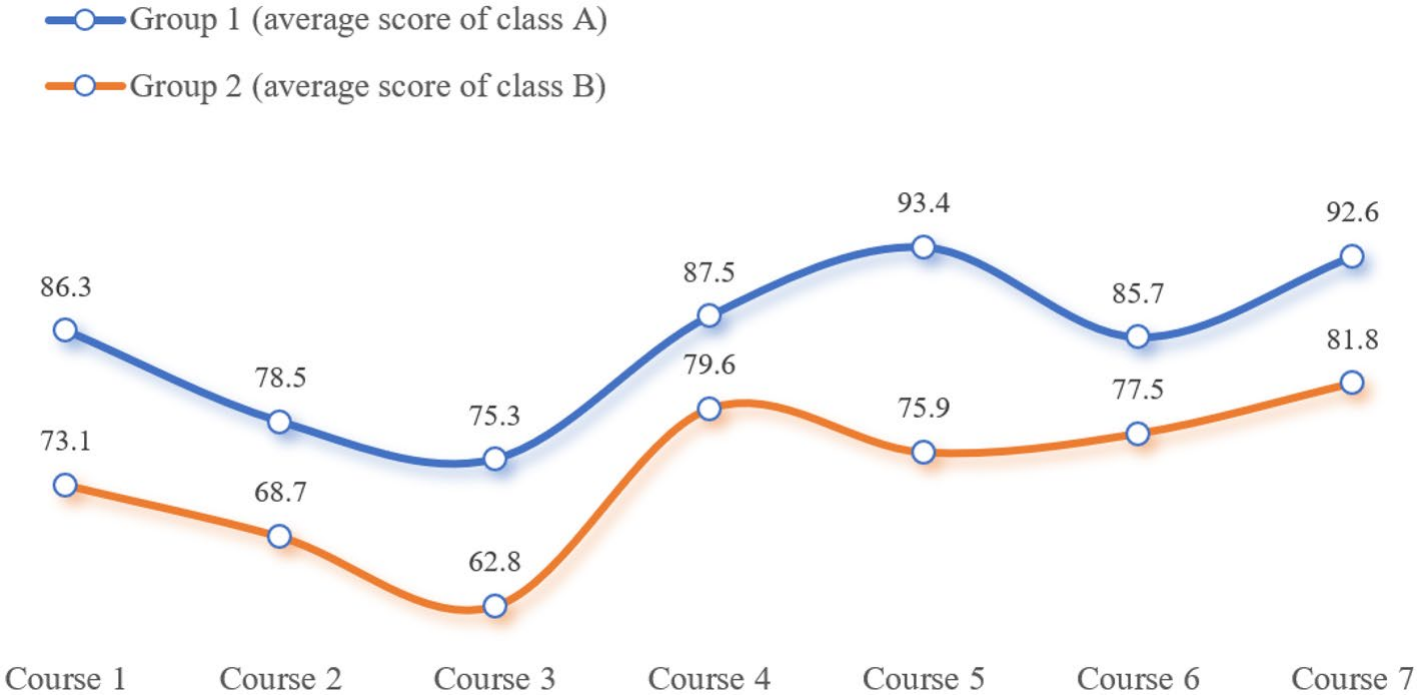
Comparison of core curriculum grades for two sample groups.

**Figure 19:**
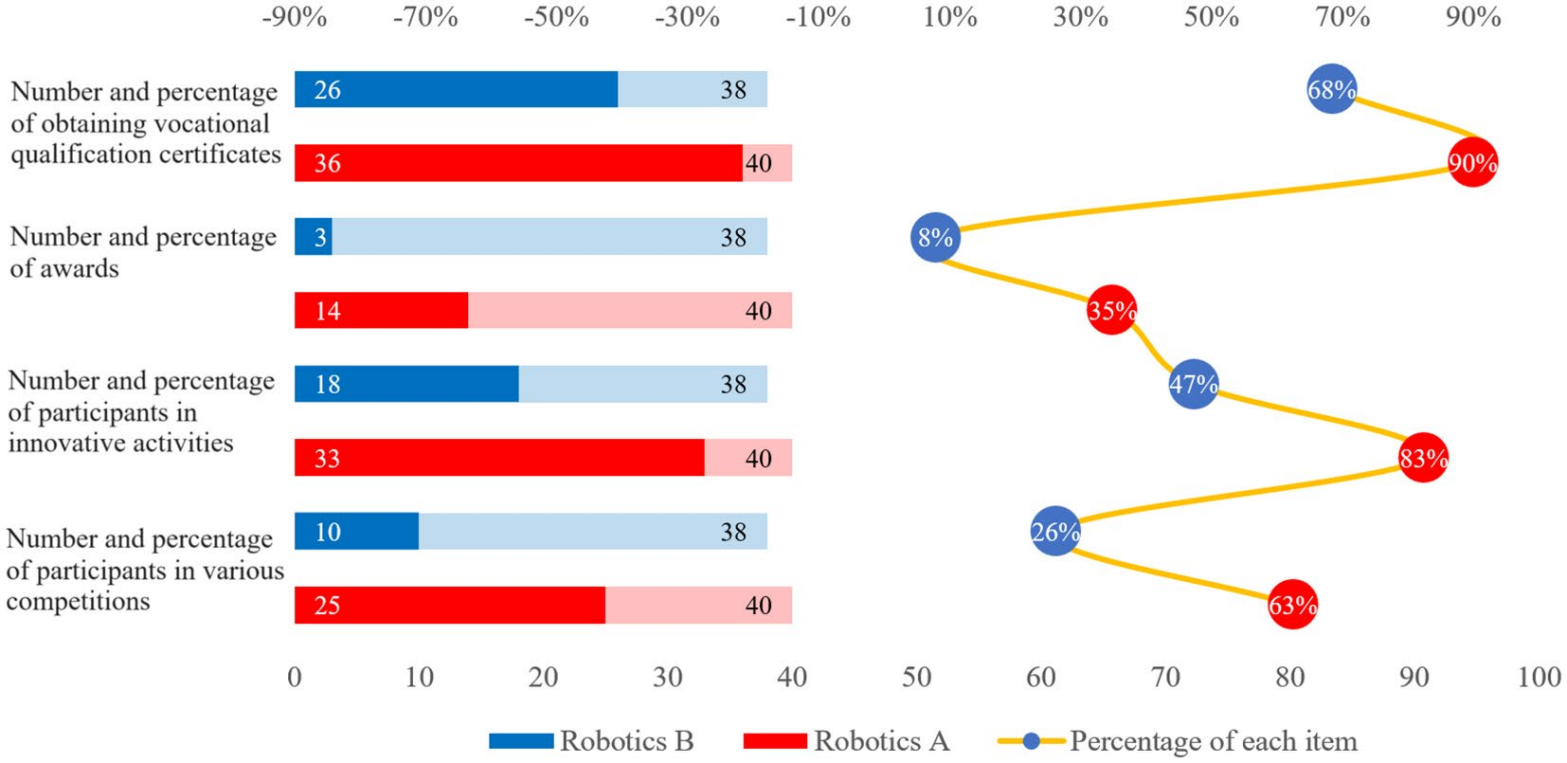
Comparison of the performance of the industrial robotics on other academic indicators.

According to the teaching effect comparison scheme, the degree of mastery of professional core knowledge of the two groups of students is quantitatively compared and analyzed in the form of grades, and the actual effect of the two teaching modes is comprehensively evaluated in different dimensions, such as the grades of the core courses in one academic year of their major, the proportion of the class participating in the various college students' vocational skills competitions and activities to improve their innovation ability and the number of awards, and the rate of certificates obtained by the class participating in the vocational qualification level exams, and so on. As shown in Fig. 18, it is shown that: after practical comparison, the overall learning effect of the first group of different majors is significantly better than that of the second group, and this group is more outstanding in terms of professional core competence, enthusiasm for participation, innovation and practice. By comparing the six samples of three different majors under the intelligent manufacturing category in terms of their professional core course scores (Electrical Automation: PLC Motion Control Technology (Course 1), Industrial Robot Field Programming (Course 2); Industrial Robotics: Industrial Robot Assembly and Commissioning (Course 3), Touch Screen Human–Machine Interface Technology (Course 4), Industrial Robot Application System Integration (Course 5); Mechatronics: PLC Application Technology (Course 6), Hydraulics and Pneumatics Technology (Course 7)) and related academic index points, it can be concluded that: the average scores of one academic year professional core course scores of the three samples of Automation A, Mechatronics A and Robotics A in the first group are higher than those of the samples of Automation B, Mechatronics B and Robotics B of the second group by 11.5, 9.5 and 12.6 points respectively. In addition, the proportions of the three samples A in the first group, who participated in various competitions, innovative ability enhancement activities, won prizes in competitions and obtained vocational qualification level certificates were significantly higher than those of the three samples B in the second group. As shown in Fig. 19, take the Industrial Robotics major as an example, the four indexes of the samples in the first group were higher than those of the samples in the second group by 37%, 36%, 27% and 22%, respectively.

Consequently, the application practice of this virtual simulation teaching platform shows that the virtual simulation teaching platform established in this paper for intelligent manufacturing has a greater effect on the practical teaching effect of engineering, which can significantly improve the teaching dilemma of the tight experimental equipment in the institutions, significantly increase the average practical operation opportunities, effectively stimulate the students' interest in learning, and improve the comprehensive academic level of the students. Through the construction of professional virtual simulation teaching platform, it helps to use the new generation of information technology such as virtual reality, digital twin, develop resources, upgrade equipment, build courses, form teams, innovate the traditional practice teaching mode, effectively serve the professional practical training and social training, and enhance the cultivation ability of the local institutions for the intelligent manufacturing composite talents.

Nevertheless, due to the limited construction sites and funds, the construction program proposed in this paper does not cover all similar majors under the major category of equipment manufacturing, and the virtual simulation modules of some core courses are not embodied, such as the virtual simulation content of sensors and intelligent detection technology. Additionally, this work only takes three majors in the field of intelligent manufacturing as teaching practice samples, which is inevitably slightly insufficient in the test sample size and the representativeness of the sample group, which will bring challenges and limitations to the universality and wide promotion of this study. With the expansion of the experimental site of the researcher's college and the advancement of the construction process of the new campus, we will plan and build a larger-scale modern engineering practice center in the new campus, striving to be built as a national virtual simulation training teaching base, incorporating the second phase of the virtual simulation teaching project, covering the content of the virtual simulation teaching of intelligent sensors, intelligent detection, robotic welding, multi-axis complex machining, collaborative robots, and opto-machine-electrical integration, and others. On this basis, we will join hands with more similar institutions in the region in the future, in the form of setting up a new engineering education alliance, to jointly carry out applied research on practical teaching reforms for students majoring in equipment manufacturing and electronics and information technology in more universities.

The virtual simulation teaching platform design ideas proposed in this paper have positive significance for improving the traditional engineering practice teaching methods, especially the design logic of constructing a shared teaching platform of virtual simulation with a major category or a professional group as a carrier has obvious superiority for perfecting the curriculum system, enriching the teaching methods, and reducing the duplication of inputs. This Mooc-like open practice pedagogy and virtual simulation resources will provide educators and educational institutions with practical construction ideas and programs that will help break down the barriers to engineering practice teaching. Engineering practice teaching equipment is generally high cost, and the virtual simulation teaching method is just the right solution to this dilemma, we proposed the construction of similar professional sharing virtual simulation platform ideas to the educational counterparts presented a good teaching reform proposals and practice attempts.

## Conclusions

This study aims at the "high investment, high loss, high risk", "difficult to implement, difficult to observe, difficult to reproduce" and other real problems in the teaching of intelligent manufacturing professional training, combined with the actual situation of the institution, an intelligent manufacturing professional virtual simulation teaching platform is designed, constructed and applied to teaching practice. Teaching practice shows that: the virtual simulation teaching platform of intelligent manufacturing proposed in this paper can better stimulate students' learning interest, enhance students' skill level, and comply with the industrial development demand, and the virtual simulation teaching platform developed based on the school-enterprise cooperation mode is closer to the market demand and can be referred to more highly, and has made some contributions and innovative practices in the following.

1. Design the corresponding virtual simulation teaching module for the basic knowledge (such as electrical technology, electrical control and PLC, etc.) of intelligent manufacturing majors. Through the virtual simulation of basic experiments of electronics and PLC, it realizes the functions of simulation measurement of electric signals, virtual wiring and debugging simulation of PLC control units. It serves the basic experimental teaching of intelligent manufacturing specialties;
2. For the core knowledge in the intelligent manufacturing, design a typical automated production line of virtual and real combination of practical training teaching module, through the mechanical transmission, PLC, inverter, touch screen, stepping system, servo system, sensor system, etc. object modeling, to achieve the production of materials in the production chain of transmission, sorting, handling, palletizing and other actions such as the implementation of the virtual simulation and detection. The utilization of the combination of virtual and real control methods to realize the two-way interoperability and control of the virtual simulation system and the physical experimental platform not only improves the students' practical opportunities and teaching effect, but also enhances the experimental experience and authenticity;
3. For the application field of industrial robots, the virtual simulation teaching module of industrial robot virtual disassembly and two-axis collaborative robot workstation is designed, and the virtual simulation module of industrial robot mechanical transmission, electrical control and other components is constructed through the ontological modeling of the core carrier (industrial robots) in the field of intelligent manufacturing. On this basis, build a cooperative robot workstation consisting of six units, such as loading unit, processing unit, packaging unit, testing unit, sorting unit, etc., and use digital twin technology to establish its corresponding digital twin training module, to achieve the experimental effect of "virtual and real synchronization" of the physical workstation and digital prototype;
4. For the typical production scenarios in the field of intelligent manufacturing, a virtual simulation system applicable to the flexible production line of the magnetic yoke shaft of micro special motors is established in the form of school-enterprise cooperation. For students to use intelligent manufacturing professional knowledge to solve practical production problems and perception of manufacturing workshop complete process chain involved in the comprehensive knowledge provides a good on-campus teaching environment, but also fully demonstrates the virtual simulation teaching platform in solving the production of practical training teaching faced by the "difficult to implement, difficult to observe, difficult to reproduce" on the problem of great superiority.

The work proposes a design scheme of virtual simulation teaching platform for intelligent manufacturing class, and completes the construction of relevant laboratories and teaching reform practice in the author's institution, but the work carried out in the development of teaching standards around the virtual simulation class of intelligent manufacturing professional courses is still lacking. Consequently, in the future research will be put on the expansion of the national virtual simulation training teaching base, as well as the virtual simulation teaching resources of regional institutions. Integration of intelligent manufacturing class in a professional group, the joint development of intelligent manufacturing class virtual simulation teaching standard and promotion of multiple institutions. Simultaneously, a broader evaluation study on learner experience will be conducted using a standardized questionnaire for multiple universities in order to obtain broadly comparable results in the future. This may also be a new opportunity and trend to respond to the times, iterate on new technologies, and incorporate new ideas, concepts, and advances in industry and education into the development of higher education.

## Data Availability

The data used to support the findings of this study are available from the corresponding author upon request.
